# Breast MRI segmentation for density estimation: Do different methods give the same results and how much do differences matter?

**DOI:** 10.1002/mp.12320

**Published:** 2017-07-25

**Authors:** Simon J. Doran, John H. Hipwell, Rachel Denholm, Björn Eiben, Marta Busana, David J. Hawkes, Martin O. Leach, Isabel dos Santos Silva

**Affiliations:** ^1^ Division of Radiotherapy and Imaging, The Institute of Cancer Research Cancer Research UK Cancer Imaging Centre London SM2 5NG UK; ^2^ Department of Medical Physics and Bioengineering UCL, Centre for Medical Image Computing (CMIC) London WC1E 7JE UK; ^3^ Department of Non‐Communicable Disease Epidemiology London School of Hygiene & Tropical Medicine London WC1E 7HT UK

**Keywords:** ALSPAC, breast cancer, mammographic density, MRI, segmentation

## Abstract

**Purpose:**

To compare two methods of automatic breast segmentation with each other and with manual segmentation in a large subject cohort. To discuss the factors involved in selecting the most appropriate algorithm for automatic segmentation and, in particular, to investigate the appropriateness of overlap measures (e.g., Dice and Jaccard coefficients) as the primary determinant in algorithm selection.

**Methods:**

Two methods of breast segmentation were applied to the task of calculating MRI breast density in 200 subjects drawn from the Avon Longitudinal Study of Parents and Children, a large cohort study with an MRI component. A semiautomated, bias‐corrected, fuzzy C‐means (BC‐FCM) method was combined with morphological operations to segment the overall breast volume from in‐phase Dixon images. The method makes use of novel, problem‐specific insights. The resulting segmentation mask was then applied to the corresponding Dixon water and fat images, which were combined to give Dixon MRI density values. Contemporaneously acquired T_1_‐ and T_2_‐weighted image datasets were analyzed using a novel and fully automated algorithm involving image filtering, landmark identification, and explicit location of the pectoral muscle boundary. Within the region found, fat‐water discrimination was performed using an Expectation Maximization–Markov Random Field technique, yielding a second independent estimate of MRI density.

**Results:**

Images are presented for two individual women, demonstrating how the difficulty of the problem is highly subject‐specific. Dice and Jaccard coefficients comparing the semiautomated BC‐FCM method, operating on Dixon source data, with expert manual segmentation are presented. The corresponding results for the method based on T_1_‐ and T_2_‐weighted data are slightly lower in the individual cases shown, but scatter plots and interclass correlations for the cohort as a whole show that both methods do an excellent job in segmenting and classifying breast tissue.

**Conclusions:**

Epidemiological results demonstrate that both methods of automated segmentation are suitable for the chosen application and that it is important to consider a range of factors when choosing a segmentation algorithm, rather than focus narrowly on a single metric such as the Dice coefficient.

## 1 Introduction

Mammographic density, a quantitative measure of radiodense fibroglandular tissue in the breast, is one of the strongest predictors of breast cancer risk. Women with more than 75% density have a fourfold or higher risk of breast cancer compared to those with less than 5%.^1^ More intensive screening for women with high mammographic density has been proposed^2^ but remains controversial.^3^

However, in clinical practice, mammographic density, as assessed on x‐ray mammograms, is generally reported using only qualitative, radiologist‐assessed categories, and agreement between radiologists tends to be only moderate.^4^ Quantitative analysis is hampered by the fact that breast density is an inherently 3‐D material property and therefore not well suited to measurement using 2‐D x‐ray projections. Although subsequent risk assessment and epidemiological analysis rarely use full 3‐D information (normally preferring a single number, i.e., the volume‐averaged mean breast density), accurate derivation of such a statistic from the 2‐D x‐ray data is problematic and subject to error. Automated tools, such as Volpara (VolparaSolutions, Wellington, NZ)^5^ and QUANTRA (Hologic Inc., USA), are gaining traction in the mammography community, suggesting that mean breast density can be calculated without inter‐reader bias. However, such readings may be affected by errors in estimating breast thickness^6^ and the relation between the values of breast density reported and those obtained by other techniques remains to be elucidated.^7^

Increasingly, magnetic resonance imaging (MRI) mammography is being used in clinical and research settings to assess breast structure, because of its 3‐D capabilities, its nonionizing nature and the strong soft tissue contrast between fibroglandular (parenchymal) and fatty tissue. In an MRI context, breast density refers to the percentage of breast tissue volume that is deemed to be “parenchymal” and this is generally assumed to be the same as volume fraction of tissue whose MR signal arises from free water molecules (i.e., the “water fraction” or “percentage water”), as opposed to fat. Clearly, this is not an exact equivalent of the mammographic x‐ray density. Nevertheless, Thompson et al.^8^ demonstrate a clear correlation between the two.

At present, manual evaluation of MRI 3‐D breast density is an arduous, observer‐dependent, and time‐consuming process. Therefore, full or partial automation of the 3‐D analysis of the breast is required. To achieve the desired segmentations of breast parenchymal volume and breast fat volume, two separate image processing tasks are required. First, the breast as a whole needs to be distinguished from the background and chest wall; and, second, the parenchymal tissue within the breast needs to be distinguished from fat.

Several different MRI pulse sequences have previously been used to assess breast density, but no definitive consensus has been reached about which is optimal. Few studies have compared different sequences within the same subject population. Furthermore, while there is a large body of prior literature (see Table 1) describing different ways to achieve the two segmentation tasks described above, no studies, to date, have compared different automated methods with each other and with manual segmentation, for a sizeable subject population.

**Table 1:** Summary of journal papers describing methods to segment pectoral muscle and internal fibro‐glandular tissue from MR images. *N*_*OB*_ refers to the number of observers who provided the gold standard manual segmentation. *N*_*D*_ indicates the number of MR data sets the method was validated with and *N*_*S*_ the number of MRI scanners. N/A = not applicable; N/S = not specified

Author, year	Ref. no.	Breast outline segmentation method	Fat/water classification method	*N*_Obs_	*N*_*D*_	*N*_*S*_
Image processing methods						
Hayton et al. (1997)	9	Threshold, morphological opening followed by “dynamic programming”	None	N/S	3	N/S
Twellmann et al. (2005)	10	Median filtering; Otsu automated thresholding; morphological closing	None	N/S	12	1
Koenig et al. (2005)	11	Histogram‐based threshold for breast air, then Gaussian smoothing; intensity threshold for pectoral boundary, then min and max of locations with transition within confidence interval	None	N/S	4	N/S
Yao (2005)	12	Threshold, morphological opening, and region‐growing followed by Bernstein‐spline and active contour; automatic identification of key points to define rough surfaces of pectoral muscle; successive refinement via gradient‐based technique, Bernstein spline, and active contour	Fuzzy C‐means	1	90	N/S
Lu et al. (2006)	13	Region‐growing, then spline and active contour for breast‐air boundary; location of key points by geometry; identification of muscle slab, followed by spline	None	N/S	1	1
Giannini et al. (2010)	14	Region‐growing, then spline and active contour	None	2	12	2
Wang L et al. (2012)	15	Hessian sheetness filter; 3‐D connected component algorithm; intensity‐based region‐growing based on seed points automatically selected	None	1	84	5
Wu et al. (2012a,b, 2013a,b)	16, 17, 18, 19	Thresholding, morphological opening, contour extraction; three edge maps generated from original data and two nonlinear filters; candidate selection; median filtering; dynamic time‐warping; comparison between slices	Continuous Max‐Flow	1	60	4
Atlas‐based methods						
Gubern–Mérida et al. (2011)	20	Manually created atlas with 7 tissue classes; landmark detection	Bayesian atlas plus Markov random field regularization	1	27	1
Gubern‐Mérida et al. (2012), (2015)	21, 22	Manually created atlas; sternum detection; N3 bias‐field correction	EM algorithm with Gaussian mixture model	3,4	27+23	1
Gallego‐Ortiz and Martel (2012)	23	Atlas created from Dixon in‐phase images via entropy‐based groupwise registration; maximal phase congruency and Laplacian mapping	None	N/S	500	1
Khalvati et al. (2015)	24	Atlas created by manual initialization of active contour algorithm, subsequently corrected manually	None	N/S	400 + 17	3
Gallego and Martel (2011)	25	Atlas, statistical shape model	None	N/S	415	N/S
Neural networks and fuzzy C‐means						
Ertas et al. (2006), (2008)	26, 27	Breast air boundary: threshold; chest‐wall: four cascaded cellular neural networks		1	39	N/S
Wang C‐M et al. (2008)	28	Support vector machines	Support vector machines	N/S	N/S	1
Wang Y et al. (2013)	29	Support vector machines acting on multiple sets of MR images with different contrast	Support vector machines	N/S	4	1
Klifa et al. (2004), (2010)	30, 31	Fuzzy C‐means	Fuzzy C‐means	> 1	30	N/S
Yang et al. (2009)	32	Kalman filter‐based linear mixing; fuzzy C‐means	Kalman filter‐based linear mixing	N/S	1	1
Nie et al. (2008)	33	Fuzzy C‐means; V‐cut; skin‐exclusion; B‐spline; manual refinement via GUI	Fuzzy C‐means	3	11	1
Sathya et al. (2012)	34	Fuzzy C‐means; support vector machines	None	N/S	1	1
Lin et al. (2011)	35	Fuzzy C‐means and B‐spline fitting, building on,^33^ with inhomogeneity correction via N3	Fuzzy C‐means, typically with 6 clusters	1	30	1
Lin et al. (2013)	33, 36	Template‐based	As per^35^	1	30	1
Ertas et al. (2016)	37	Bias‐corrected FCM, followed by morphological opening and closing	None	1	82	> 4
This study		Bias‐corrected FCM vs thresholding, landmark analysis	Dixon vs T1w and T2w contrast	3	200	1

It is clear that many methods can produce “good” segmentation results. This study poses the following question: Do the minor differences we see between segmentations when we apply different algorithms on the same data actually matter for the uses to which the segmentations are ultimately put?

This study compares two very different methods of breast‐outline segmentation: (a) an established^37^ bias‐corrected fuzzy C‐means (BC‐FCM) clustering technique based on a cost‐function; and (b) a new heuristic approach based on thresholding, landmark identification, and direct analysis of image features. The results of this part of the study will be measures of overall breast volume from each method and volume similarity measures (Dice and Jaccard coefficients).

With the breast outline obtained, the second part of the study compares two methods of fat–water discrimination, again based on different principles. (a) The Dixon approach^38^ uses scans acquired with an MRI technique that returns separate “fat” and “water” images. In principle, these allow us to obtain a fat and water fraction for every voxel, accounting for partial volume effects. However, Dixon sequences are not currently part of the routine acquisition protocol for clinical MRI examinations.^39^ (b) Our second method uses an analysis of the intensity histograms of the two different tissue classes in fat‐suppressed *T*_1_‐weighted (T1w) and *T*_2_‐weighted (T2w) images. Such images are routinely acquired in diagnostic scanning and this method thus has the potential advantage of wider applicability if the two methods are shown to be concordant. Note that there is no means of obtaining ground truth data and, given that we are dealing with a healthy subject cohort, no possibility of obtaining x‐ray data for comparison.

**Figure 1:**
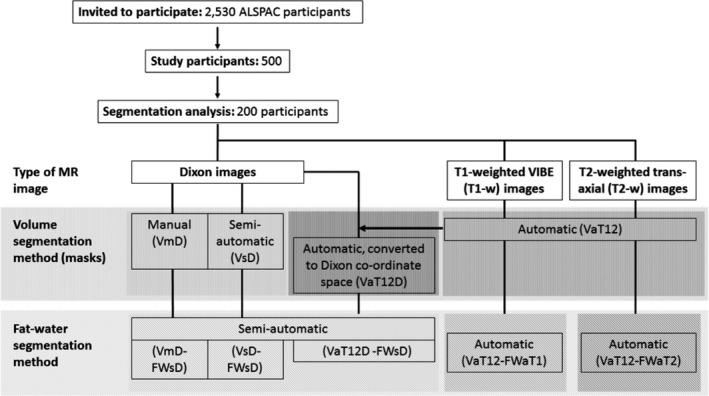
Flow diagram of the overall data processing chain and nomenclature for the various segmentation methods. Some of these have the potential to operate on different source data and we can also combine the methods in different ways to achieve an overall result. We thus assign each step three codes: *segmentation purpose* (V = breast volume, FW = fat–water); *degree of automation* (m = manual, s = semi‐automatic, a = fully automatic); and *source data* (D = Dixon; T1 = *T*_1_‐weighted, T2 = *T*_2_‐weighted, T12 = uses both *T*_1_‐ and *T*_2_‐weighted data). Thus, a breast‐volume measurement using semiautomatic segmentation on original Dixon data would be represented as VsD. Fat–water segmentations require both source data and a previously generated volume mask, so are represented by the combination of two codes. For instance, fat–water statistics calculated semiautomatically from Dixon source data and using a mask generated automatically from T1w and T2w data would be described by VaT12‐FWsD. We note one additional case, in which the volume mask VaT12 is re‐sampled to give a result in the same coordinate space as the Dixon images and we assign this the label VaT12D.

Nomenclature for the various segmentations is summarized in Fig. 1.

A comprehensive epidemiological analysis of the relationship between breast composition and seven other physical, historical and lifestyle variables has been carried out for this cohort. While the full report is beyond the scope of this study, we summarize the results and use them to discuss quantitatively the impact of differences between the various assessment methods on conducting reliable clinico‐epidemiological studies.

## 2 Methods

### 2.A. Data

#### 2.A.1. Study population

This work forms part of an investigation into breast composition at young ages, nested within the Avon Longitudinal Study of Parents and Children (ALSPAC). ALSPAC originally recruited 14,541 pregnant women resident in Avon, UK with expected dates of delivery from 1 April 1991 to 31 December 1992, as described by Boyd et al. in a cohort profile paper.^40^ For this substudy, Caucasian nulliparous women were invited to attend an MRI examination at the University of Bristol Clinical Research and Imaging Centre (CRIC) between June 2011 and November 2014. Women were restricted to those from a singleton birth, who had never been diagnosed with a hormone‐related disease, and had regularly participated in follow‐up surveys, including completing the age 20 y questionnaire (2010–2011). Of the 2530 invited, 500 (19.8%) eligible women attended.

The ALSPAC Law and Ethics Committee, and the Local Research Ethics Committees gave ethical approval for the study. The study website contains details of all the data that are available through a fully searchable data dictionary.^41^

#### 2.A.2. MR imaging

Participants underwent a breast MRI scan using a 3T Siemens Skyra MR system with a breast coil that surrounds both breasts of a prone patient. Three sets of bilateral images were acquired:

- multislice, sagittal Dixon^38^ images (in‐phase, out‐of‐phase, water and fat), acquired using a turbo spin‐echo sequence with nominal in‐plane resolution of (0.742 × 0.742) mm^2^, nominal slice thickness 7 mm and interslice spacing 7.7 mm;
- T1‐weighted 3D images, acquired using a VIBE sequence with fat saturation and a nominal resolution of (0.759 × 0.759 × 0.900) mm^3^, as routinely used in clinical dynamic contrast‐enhanced MRI protocols for the breast;
- multislice, axial, T2‐weighted images, acquired using a turbo spin‐echo sequence, with nominal in‐plane resolution of (0.848 × 0.848) mm^2^, and both slice thickness and spacing between slices 4 mm.

#### 2.A.3. Manual reference segmentation

To assess breast volume, a manual segmentation protocol (as described in the Supplementary Information) was developed and used by three readers (RD, MB, and ISS) independently to outline the breast from surrounding tissues in the Dixon images, using ITK‐SNAP (version 3.0.0). All subjects had a manual segmentation of all breast slices performed by at least one reader. The datasets of 16 representative subjects were manually segmented twice by all three readers to assess between‐ and within‐observer variation. In cases where more than one manual segmentation is performed, the VmD and VmD‐FWsD results quoted below represent the median values taken for the multiple manual readings.

#### 2.A.4. Training and validation data sets

A training set of 100 randomly selected subjects was used to make initial comparisons across MR images and segmentation methods, and for the manual readings, between‐ and within‐observer variation. The training data were used to assess the common reasons for segmentation failure and to improve the algorithms. At the end of the testing phase, the algorithm code was “frozen” and final comparisons of the segmentation methods were completed on a second set of images from a further 100 participants. Except where stated otherwise, all the summary statistical results presented here come from this second, “validation” cohort. For further details concerning statistical methods, please see the Supplementary Information.

### 2.B. Breast outline segmentation

#### 2.B.1. Semiautomated, bias‐corrected fuzzy C‐means (BC‐FCM)

A fuzzy C‐means (FCM) algorithm was applied to the Dixon in‐phase images. It has the advantage that it can be modified to carry out a simultaneous intensity inhomogeneity compensation, or bias‐correction (BC), and this is potentially less expensive computationally than a prefiltering operation.^42^ The algorithms in this section were implemented using IDL (Harris Geospatial Systems, Melbourne, FL, USA) and run on a standard desktop computer.

The BC‐FCM variant we implemented is described in.^37^ Formally, the algorithm does not require a training dataset and so is an unsupervised clustering algorithm. However, in practice, some experience with the types of data involved can improve the results dramatically. Except for the local smoothness criterion (introduced by cost function *γ* in ref. 37 — see this publication for all other related notation), BC‐FCM *per se* does not use any spatial information. Nevertheless, a “good” segmentation involves a number of problem‐specific insights and the basic BC‐FCM method above was enhanced by additional heuristic algorithms in the spatial domain, based on the results obtained with the training data.

##### Initial parameters and iteration threshold

After some experimentation, *β*(**r**) was set to 0.1 for all spatial locations and *ε* to 0.01. The two initial class centroids *c*_*f*_ were calculated by taking the mean of the slice being processed and adding a lower and an upper offset. These two offsets are adjustable parameters under user control. For many subjects (see the Results section for an example), a single set of defaults performed extremely well. However, for a small subset of “difficult” cases (second example in Results), user interaction was needed to try various combinations. As implemented here, on a standard desktop computer, running nonoptimized software, it took around 2 min to run the segmentation algorithm on each 3‐D dataset. Thus, this “trial and error” step was the most frustrating feature of the BC‐FCM method in practice. Numerous coding and hardware improvements (e.g., parallelization) could be made to the prototype to improve the user experience, potentially allowing these adjustable parameters to be altered by simple slider controls with immediate feedback.

We observed an improvement in performance by allowing the algorithm to perform separate BC‐FCM classifications for segmenting the posterior of the breast from the chest wall and segmenting the anterior portion from air, then merging the two volumes. Furthermore, it was noted that the optimal offsets providing the initial class centroids were often different for these two segmentation problems. Thus, each dataset is split into two portions in an anterior‐posterior (AP) direction and the BC‐FCM algorithm applied twice per image slice. Given that the size of breasts varies, the position of the AP‐split is also different for different datasets and this is handled automatically by having two passes through the entire algorithm with an automated choice of the AP‐split position made after Pass 1.

##### Morphological operations

The breast outlining task requires a definite boundary to be drawn. Thus, it is not necessary to use the full membership function output of the BC‐FCM routine, and we arrange for the clustering to produce a binary image. This may include some misclassified regions outside the breast and some “holes” inside the breast. To remove the unwanted regions, 2D hole‐filling followed by a 4‐neighborhood connectivity search and object labeling is performed. The largest nonbackground object in each slice is identified as the breast region and other smaller objects are removed from the binary image. This exercise is repeated for all slices and these are then merged to form an approximate breast volume.

Within this approximate breast volume, there may be some nonbreast tissue segmented for cases in which fatty breast tissue is connected to the chest and liver; and there may also be some unsegmented breast tissue left for cases in which dense breast tissue is connected to the chest wall muscles. To reduce these over‐ and undersegmentations, 3D morphological image opening is performed, followed by closing using two cylindrical structuring elements having the same radius of 3 voxels but different heights of 3 voxels and 25 voxels in the axial direction. These parameters were found by experimentation during our previous study.^37^

##### Lateral cutoffs

The preceding steps in the process do an excellent job in segmenting the anterior and posterior margins of the breast. However, there is no consensus in the literature as to “where the breast stops” in the right‐left and superior‐inferior directions. The extent of the breast is not directly delineated by any change in MRI contrast and the required boundary may, indeed, be specific to the application of the imaging (e.g., when comparing the MRI segmentation with the breast region compressed within the paddles of a mammography system, the axilla region may be excluded entirely). Thus, based on the consensus protocol (Appendix S1) reached by the three experienced readers, a heuristic algorithm was developed, as described below. This additional truncation is derived entirely from geometric considerations and boundaries are drawn without regard to image intensity, which is in many cases the same on either side of the boundary.

Each breast is processed in turn. The stack of sagittal images segmented using BC‐FCM forms a pseudo 3‐D dataset. From this dataset, the transverse plane containing the largest breast area is passed to a simple algorithm that extracts the air‐breast interface as a 1‐D “breast profile”. (This geometry is illustrated as Figure S2 of the Supplementary Information.) The profile is used to determine the position of the breast midpoint in a left‐right direction. Working outwards from this midpoint, we find the first position at which the absolute value of the gradient (approximated by the finite difference between adjacent voxels) of the breast profile rises above a threshold value, determined by experimentation. This indicates a change in angle of the skin surface from flat regions between and outside the breasts, to the side contour of the breast. A mask is applied to exclude all sagittal slices in the original dataset on either side of these changes in angle. (Typically, the “raw” output of the BC‐FCM algorithm would include these.) Finally, a similar profile is generated for the superior‐inferior direction and the upper and lower bounds of the breast are determined in each sagittal plane of the original data.

#### 2.B.2. Fully automated, using T1w and T2w images

##### Preprocessing processing (bias‐field correction)

**Figure 2:**
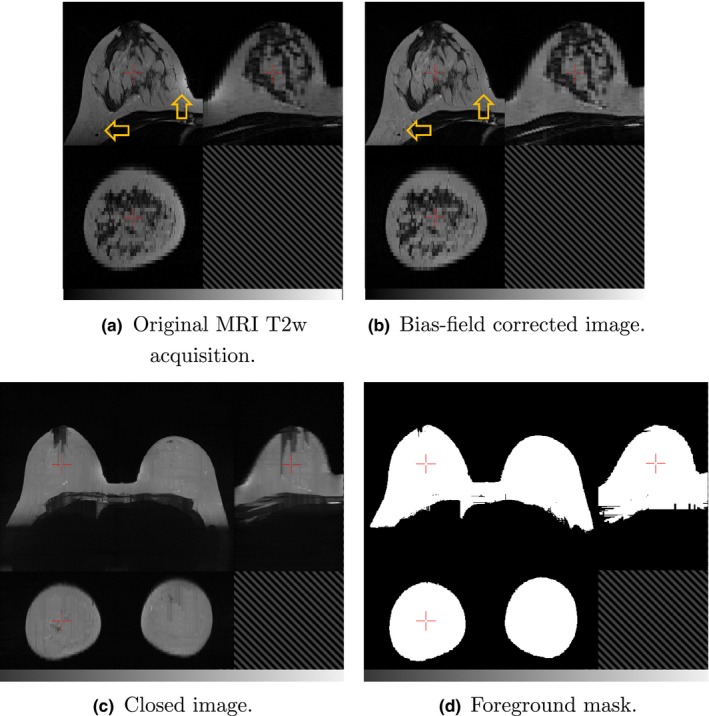
Orthogonal slices through (a) a T2 weighted MRI and (b) the corresponding image after bias‐field correction, with arrows indicating regions that are particularly improved by the processing. The “closed” T2w image is shown in (c) and foreground mask *I*_fg_ in (d). In each image, the top‐left quadrant is the axial slice, the top‐right is sagittal and the bottom‐left is coronal. [Color figure can be viewed at wileyonlinelibrary.com]

A slowly varying bias‐field, caused by inhomogeneities in the magnetic field during the MR acquisition, is a common artifact of MR images. To correct this for the T1w and T2w images, we apply the “N4ITK” nonparametric nonuniform intensity normalization method.^43^ This is a refinement of the popular N3 algorithm which adopts a fast, robust B‐spline fitting algorithm and a hierarchical, multiscale, optimization scheme [Figs. 2(a) and 2(b)].

##### Breast mask segmentation

This novel, heuristic method, implemented using the Insight Toolkit,^44^ computes a whole breast mask using both the T1w and T2w images. In developing this automated approach, emphasis has been placed on limiting the number of empirically derived parameters and relying instead on detecting statistical or functional extrema. In this way, we aim to make the method as widely applicable to variations in subjects and images as possible. The method comprises a number of distinct processing steps as follows.

**Figure 3:**
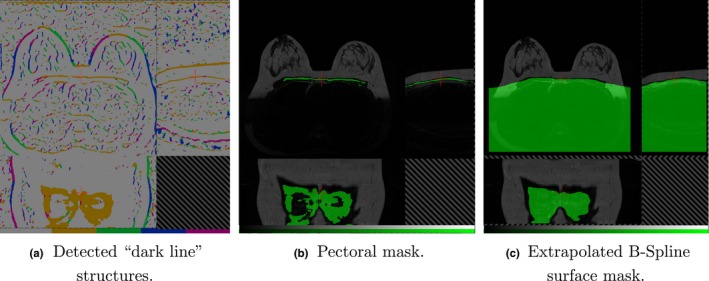
The anterior pectoral muscle surface is detected using the Oriented Basic Image Feature “dark line” class. Subplot (a) shows these features detected at four orientations (OBIF_15_ to OBIF_18_). Region growing the “brown” medial‐lateral class, OBIF_15_, closely delineates this anterior boundary immediately posterior to the sternum (b). The anterior surface of this mask is extrapolated using a B‐Spline fit to the lateral boundaries of the volume (c). [Color figure can be viewed at wileyonlinelibrary.com]

**Figure 4:**
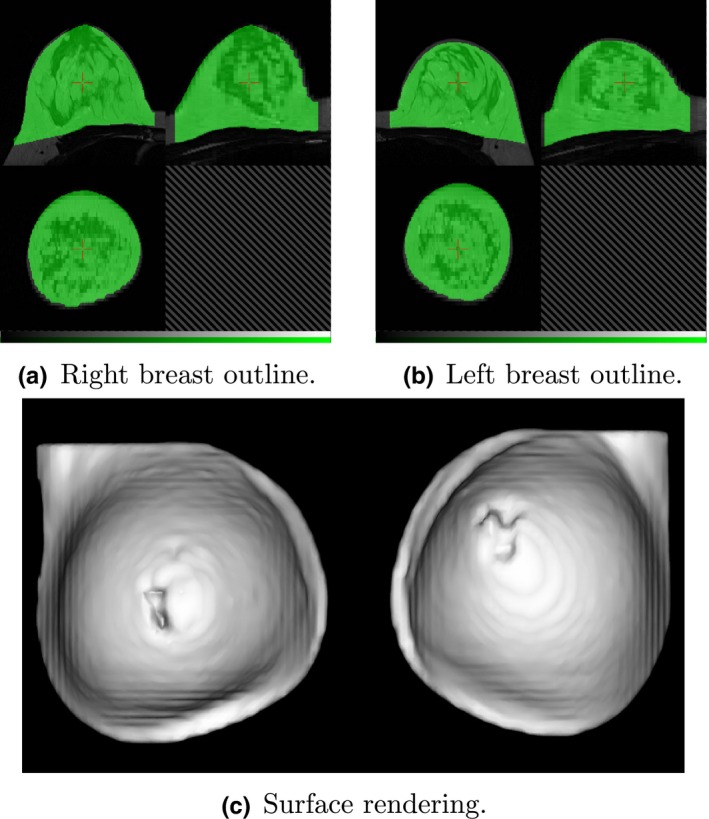
Breast region mask created by removing the pectoral surface mask (Fig. 3) from the foreground mask (Fig. 2). Two views of the mask are shown, superimposed on the original MR image and centered on the right (a) and left (b) breasts. The surface rendering (c) illustrates the “squaring off” to include the axilla. [Color figure can be viewed at wileyonlinelibrary.com]

1. The T2w image is resampled to match the resolution of the T1w image.
2. A grey‐scale closing operation along each of the orthogonal axes, **x**,** y** and **z**, is performed on the T2w image, to eliminate voids from the subsequent foreground segmentation. In this operation, each voxel's intensity, *I*_T2w_, at index (*i*,* j*,* k*) is replaced by *I*_cT2w_(*i*,* j*,* k*) according to:
  (1) $$\begin{array}{cc} I_{\mathrm{cT}2w}\left(i,\;j\;,k\right)=\text{min}\;\; & \left[\text{min}\left(\max_{0\leq i_{1}\leq i}I_{T2w}\left(i_{1},\;j\;,\;k\right),\max_{i<i_{2}<N_{i}}I_{T2w}\left(i_{2},\;j,\;k\right)\right),\right.\\ & \text{min}\left(\max_{0\leq j_{1}\leq j}I_{T2w}\left(i,\;j_{1},\;k\right),\max_{j<j_{2}<N_{j}}I_{T2w}\left(i,\;j_{2},\;k\right)\right),\\ & \left.\text{min}\left(\max_{0\leq k_{1}\leq k}I_{T2w}\left(i,\;j,\;k_{1}\right),\max_{k<k_{2}<N_{k}}I_{T2w}\left(i,\;j,\;k_{2}\right)\right)\right] \end{array}$$
  where *N*_*i*_, *N*_*j*_, *N*_*k*_ are the number of voxels along each axis.
3. The T1w image is rescaled to match the intensity range of the closed T2w image and the maximum of these two images, *I*_MaxT1wT2w_, computed.
4. The foreground (i.e., the subject) is segmented from the background by thresholding, *I*_MaxT1wT2w_. The threshold, *t*_bg_, is computed via:
  (2) $$\begin{array}{c} t_{\mathrm{bg}}=\underset{I}{\text{arg max}}\left[F_{\mathrm{dark}}\left(I\right)\left(F_{\mathrm{CDT}}\left(I\right)-F_{\mathrm{var}}\left(I\right)\right)\right] \end{array}$$
  according to the following functional criteria:
  - The background is assumed dark therefore the threshold should be close to zero:
    (3) $$\begin{array}{c} F_{\mathrm{dark}}\left(I\right)=1-\frac{I}{\max(I)} \end{array}$$
  - The frequency of voxel intensities in the background is higher than the foreground, i.e., the background intensities form a distinctive peak in the image histogram, P(I), which is captured by a sharp rise in the cumulative intensity distribution function:
    (4) $$\begin{array}{c} F_{\mathrm{CDT}}\left(I\right)=\frac{\sum_{j=0}^{I}P\left(j\right)}{\sum_{k=0}^{\max(I)}P\left(k\right)} \end{array}$$
  - The background has a lower intensity variance than the foreground:
    (5) $$\begin{array}{c} F_{\text{var}}\left(I\right)=\frac{\sum_{j=0}^{I}P\left(j\right){\left(j-\mu \right)}^{2}}{\sum_{k=0}^{\max(I)}P\left(k\right){\left(k-\mu \right)}^{2}} \end{array}$$
    The resulting foreground mask image is denoted *I*_fg_ — see Fig. 2(d).
5. Landmark identification. The most anterior voxels in the foreground mask, *I*_fg_, on the left and right sides of the volume, are identified and assumed to be approximately coincident with the nipple locations. If multiple voxels are found, then the center of mass of the cluster is computed. The midsternum is computed as the most anterior voxel of the foreground mask, equidistant from the nipple landmarks in the coronal plane.
6. Pectoral muscle boundary extraction. Various methods have been presented in the literature to segment breast MRI volumes and the pectoral muscle (Table 1). These include semiautomated methods requiring user interaction,^31^, ^33^, ^36^ 2D midslice template registration,^36^ statistical shape models,^25^ and atlas‐based methods.^16,18‐20,24,25^ A number of methods have been developed to segment explicitly the pectoral muscle. These include a B‐spline fit to the intensity gradient of the pectoral boundary,^33^ anisotropic diffusion and Canny edge detection,^17^ and Hessian matrix planar shape filtering.^15^, ^46^ Atlas‐based methods have been shown to perform well but are computationally intensive^47^ and require significant initial investment of time to develop a library of atlases.
  We have developed a method to detect explicitly the anterior pectoral muscle boundary in individual MR volumes. Our approach has similarities to the Hessian processing of Wang et al.,^15^, ^46^ in that it employs Gaussian derivatives to detect regions in the image with a planar profile. However, rather than computing a ratio of the eigenvalues of the Hessian matrix and thresholding the result, we obtain a direct classification of linear structures, immediately posterior to the sternum, using Oriented Basic Image Features (OBIFs, Fig. 3).
  concept of Basic Image Features (BIFs) was developed by Griffin.^48^ The technique classifies pixels in a 2D image into one of seven classes according to the local zero‐, first‐, or second‐order structure. This structure is computed using a bank of six derivative of Gaussian filters (*L*_00_, *L*_10_, *L*_01_, *L*_20_, *L*_11_ and *L*_02_) which calculate the nth (where n = 0,1,2) order derivatives of the image in *x* and *y* (*S*_00_, *S*_10_, *S*_01_, *S*_20_, *S*_11_ and *S*_02_). By combining the outputs of these filters, any given pixel can be classified according to the largest component of vector BIF:
  (6) BIF=flatϵS00,slope−like2S102+S012,maximumλ,minimum−λ,lightlineλ+γ2,darklineλ−γ2,saddleγ
  given
  (7) $$\lambda =\sigma^{2}\frac{\left(S_{20}+S_{02}\right)}{2}$$
  (8) $$\gamma =\sigma^{2}\sqrt{{\left(S_{20}+S_{02}\right)}^{2}\;+\;4S_{11}^{2}}$$
  In addition, slopes, light lines, dark lines, and saddles can be characterized according to their orientation (OBIFs). We quantize this orientation into four, 45 degree quadrants which produces eight slope subclasses (OBIF_1_ to OBIF_8_), and four subclasses for each of light lines (OBIF_11_ to OBIF_14_), dark lines (OBIF_15_ to OBIF_18_), and saddles (OBIF_19_ to OBIF_22_).
  By region‐growing the medial‐lateral, OBIF_15_ dark line features detected in each axial image slice, in 3‐D, from seed positions immediately posterior to the midsternum, we obtain a binary segmentation of the anterior pectoral muscle surface. The BIF processing was performed at a single scale using a Gaussian kernel with standard deviation 5 mm. A smooth B‐spline surface is then fitted to the anterior voxels of the resulting mask^44^ to extrapolate the muscle surface to the lateral boundaries of the image volume [Fig. 3(c)].
7. Finally, we generate a 2D coronal mask, *I*_CNL_, to crop nonbreast tissue from the whole breast mask. *I*_CNL_ is computed from a coronal skin elevation map, *I*_s*kin*2*D*,_ which contains the distance of each anterior skin voxel in the foreground mask, *I*_fg_, from the most posterior boundary of the MR volume. The coronal profile of each breast is obtained by thresholding *I*_s*kin*2*D*_ at
  (9) $$\begin{array}{c} h=\frac{\left(4h_{\mathrm{ms}}+h_{\mathrm{Ln}}+h_{\mathrm{Rn}}\right)}{6} \end{array}$$
  where *h*_ms_ is the anterior elevation of the midsternum landmark, and *h*_Ln_ and *h*_Rn_ are the left and right nipple anterior elevations, respectively. The roughly circular profile obtained for each breast is then dilated by 10 mm and the mask squared off, to create a superior‐lateral corner and hence extend the breast volume into the axilla [Fig. 4(c)]

### 2.C. Fat–water discrimination

#### 2.C.1. Semiautomated calculation of percentage breast density, based on dixon images

In principle, the output from a Dixon pulse sequence is a set of images reflecting water content *I*_*w*_(**r**), which we identify with the parenchymal component of the breast, and an equivalent set *I*_*f*_(**r**) reflecting fat content. Ideally, these images would be quantitative and allow the direct calculation of the water and fat fractions *ϕ*_*w*_(**r**) and *ϕ*_*f*_ (**r**) via the equation^49^

(10) $$\phi_{w}=\frac{I_{w}}{I_{w}+I_{f}}\;\text{and}\;\phi_{f}=\frac{I_{f}}{I_{w}+I_{f}}$$

In practice, there are a number of complicating factors:

- Parenchymal tissue and fat have different relaxation properties and, since the acquisitions are not generally designed to be proton density weighted, this means that the relative intensities of equal fractions of fat and water are different.
- The *B*_1_ field of the probe is not uniform across the whole breast and this leads to a spatially dependent efficacy of the fat–water separation.
- In practice, the fat tissue does not have a single proton resonance.
- Different manufacturers have different proprietary image reconstruction methods and these may influence the quantitative results.

Our solution to (at least) the first of these problems is to proceed as follows:

- Identify a small region in the water image that is expected to be entirely composed of parenchymal tissue. The region should be in a part of the image that is free from intensity artifacts caused by proximity to the RF coil (i.e., the data should come from a homogenous region of *B*_1_).
- In the fat image, identify similarly a second region entirely composed of fat.
- Calculate the ratio of the average voxel values in each of the two regions:
  (11) $$\begin{array}{c} r\;=\;\frac{1}{N_{w}}\sum_{i\in {\mathrm{ROI}}_{w}}I_{w}\left(r_{i}\right)/\frac{1}{N_{f}}\sum_{j\in {\mathrm{ROI}}_{f}}I_{f}\left(r_{j}\right) \end{array}$$
  where *N*_*w*_ and *N*_*f*_ are the numbers of voxels in the selected regions‐of‐interest ROI_*w*_ and ROI_*f*_, respectively.
- Replace the value *I*_*f*_ in Eq. (9) with *rI*_*f*_.

This procedure potentially improves the accuracy of the water‐fraction calculation but at the cost of introducing an interactive step into the density estimation process. We have not tested in a systematic fashion the influence that the size and shape of the region‐of‐interest selection have on the process, in part because we have no ground truth values. A further issue with this technique is that in the limiting cases of extremely dense or extremely fatty tissues, it may not be possible to find appropriately “pure” regions of both types.

#### 2.C.2. Fully automated, using T1w and T2w Images

Fuzzy c‐means (FCM) clustering has been evaluated by a number of studies to classify the internal structure of the breast into fat and fibroglandular tissue classes^16,18,29,31,33‐35,50^ Table 1). Song et al.^50^ adopt a Gaussian kernel FCM, while Sathya^34^ use a quadratic kernel FCM to train a support vector machine (SVM). In,^29^ Wang et al. use a multiparametric hierarchical SVM classification approach to segment the internal breast and found this to be superior to both a conventional SVM^28^ and FCM segmentation. T1W, T2W, proton density, and three‐point Dixon (water and fat) images were all incorporated. Klifa et al.^31^ compared the resulting volumetric MRI density measurement of their method with mammography but found only modest correlation (*R* ^2^ = 0.67).

In,^20^ a probabilistic atlas approach was proposed. This requires a sizeable number of prelabeled atlases to be created, considerable computation to register them and assumes correspondence between fibroglandular structures across the population. To address the latter, a Markov random field (MRF) was introduced to spatially regularize the classification of each voxel according to that of its neighbors. Similarly, Wu et al.^16^ use the registered atlas as a pixel‐wise fibroglandular likelihood prior for a multivariate Gaussian mixture model and demonstrate superior performance when compared to FCM using a manual thresholding approach as the gold standard. In a later publication,^19^ the same authors investigate a continuous max‐flow (CMF) algorithm to generate a voxel‐wise likelihood map using the same atlas initialization. They demonstrate that this approach performs better with the atlas initialization than without, but that FCM is superior to the CMF approach without the atlas.

Mixture models have also been proposed by Yang et al.^32^ who implement a method using a Kalman filter‐based linear mixing. They demonstrate it out‐performs a c‐means method but evaluation using real MR data was limited.

Our segmentation of the T1 and T2 MRI data into fat and glandular tissue is a modification of that proposed by Van Leemput et al.^51^ in which an intensity model and spatial regularization scheme are optimized using a maximum likelihood formulation of the expectation‐maximization (EM) algorithm. The EM algorithm iteratively updates the Gaussian probability distributions used to estimate the intensity histograms of each tissue class (fat and nonfat) via a maximum likelihood formulation. In order to improve classification of voxels in which the partial volume of fat and glandular tissues is a significant factor, a Markov random field (MRF) regularization scheme is employed to ensure spatial consistency. The MRF modifies the probability of a particular voxel being assigned to either the fat or glandular classes (or a proportion of either) according to the current classification of neighboring voxels. In this way, isolated regions of glandular tissue in very fatty regions, for instance, are penalized in favor of a more realistic and anatomically correct arrangement of the classes.

### 2.D. Epidemiology

Appropriate linear and logistic regression models were used to examine associations of average total breast, fat and water volumes, and percent water, as measured using different MR images and segmentation methods, with selected established and potential mammographic density correlates. Breast measures were log‐transformed and the exponentiated estimated regression parameters represent the relative change (RC) in breast measure with a unit increase, or category change, in the exposure of interest (with 95% confidence intervals (95% CI) calculated by exponentiating the original 95% CIs). Age at menarche (months), height (cm), and BMI (height (cm)/ weight (kg)^2^) at MR were treated as continuous variables and centered at the mean. Current hormone contraceptive use, cigarette smoking, and alcohol drinking were treated as binary (yes/no) variables. Mothers mammographic density (%) was averaged between both breasts, and maternal age (months) at mammography and clinically measured or self‐reported maternal BMI (median 3 yr (interquartile range (IQR) = 1.5 yr) prior to mammography)) were used as continuous measures and centered at the mean. Variables were included as potential determinants of breast measures, or as confounding factors, where appropriate.

Data analysis was conducted with STATA statistical software, Version 14.

## 3 Results

### 3.A. Breast outline segmentation

**Figure 5:**
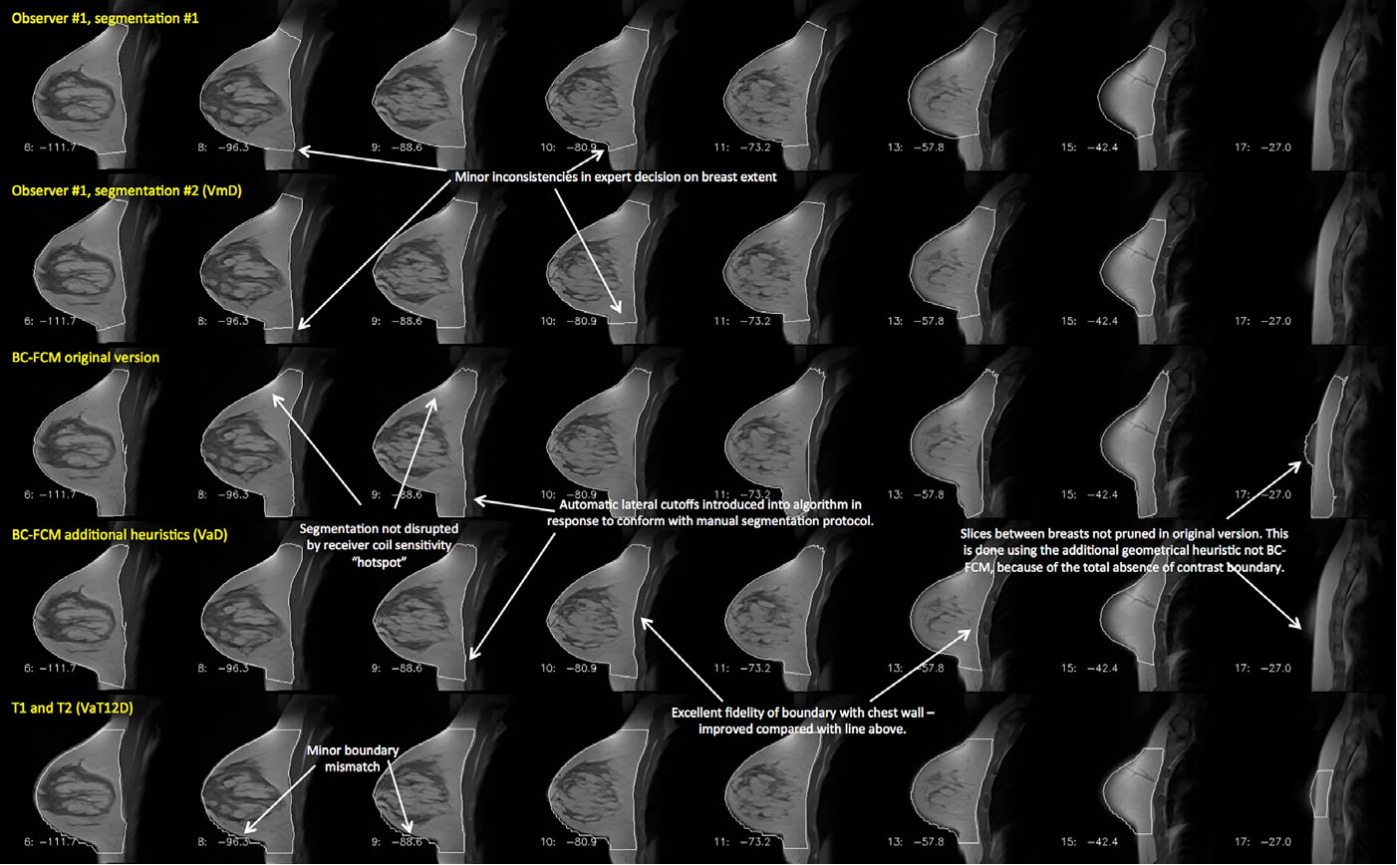
Example of a case where both of the algorithms examined in this work performed well. Features of interest in the various different segmentations are annotated. *Note that this image is provided with high resolution and can be zoomed significantly to reveal additional detail*. [Color figure can be viewed at wileyonlinelibrary.com]

Figure 5 shows an example of the two methods applied to a dataset containing medium‐sized breasts, with a moderate parenchymal content. There is a border of fat around the parenchyma, which, at the posterior of the breast, leads to excellent contrast at the boundary with the chest wall, making segmentation a relatively straightforward task. Results are shown for two separate manual segmentations by the same experienced observer; for the BC‐FCM method from ref. 37; the BC‐FCM method with additional heuristics and default parameters, as described above; and the new method based on T1 and T2 images (VaT12). It will be seen that the segmentation performance is excellent, with only minor difference between the methods. Note how implementation of guidelines developed during the manual segmentation process supplements the BC‐FCM approach in order to cut off the segmentation in both the left‐right and superior‐inferior directions, where there are no corresponding intensity boundaries seen in the image data themselves.

Table 2 shows the Dice and Jaccard coefficients for the four sets of segmentations illustrated in Fig. 5, confirming the excellent performance of all the algorithms.

**Table 2:** Dice and Jaccard coefficients for the “easy” segmentation problem of Fig. 5. Note that the BC‐FCM/heuristics (VaD) represents the fully automated version, running with default parameters

	Manual 1	Manual 2	BC‐FCM Orig	BC‐FCM /heuristics(VaD)	VaT12D
Dice coefficients					
Manual 1	1.000				
Manual 2	0.949	1.000			
BC‐FCM Orig	0.854	0.877	1.000		
BC‐FCM/heuristics (VaD)	0.901	0.924	0.921	1.000	
VaT12D	0.887	0.888	0.810	0.865	1.000
Jaccard coefficients					
Manual 1	1.000				
Manual 2	0.904	1.000			
BC‐FCM Orig	0.745	0.781	1.000		
BC‐FCM/heuristics	0.820	0.859	0.853	1.000	
VaT12D	0.797	0.799	0.681	0.761	1.000

**Figure 6:**
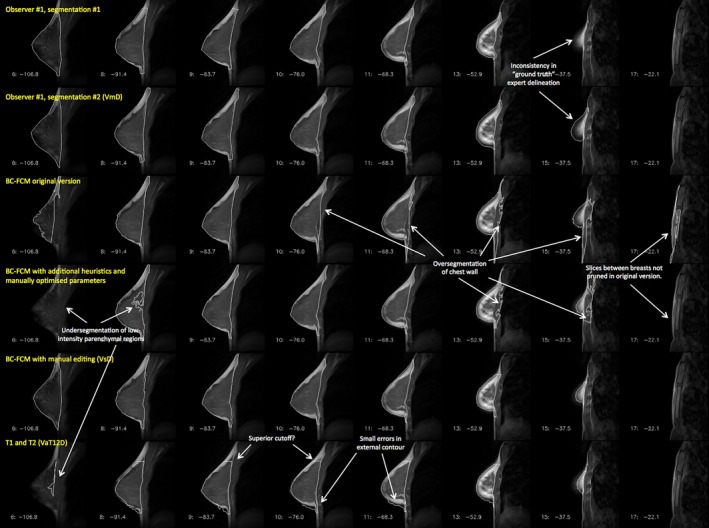
Example of a case where automatic segmentation is difficult. The rows represent the results of different segmentations and, for compactness, an informative subset of slices has been chosen to illustrate important features of the problem. *Note that this image is provided with high resolution and can be zoomed significantly to reveal additional detail*. [Color figure can be viewed at wileyonlinelibrary.com]

By contrast, Fig. 6 illustrates a case where all assessment methods have far more difficulty in providing a correct segmentation. Smaller breasts tend to be more problematic to segment, as a higher fraction of the segmentation involves partial‐volume effects. Highly parenchymal breasts have very low (sometimes no) contrast between the parenchyma and pectoral muscles of the chest wall, and the intensity‐based BC‐FCM algorithm has particular difficulties in this regard. Many slices require a high degree of anatomical knowledge to perform the segmentation. Consider the two versions of the BC‐FCM results presented. With the default parameters in the upper of the two rows, oversegmentation occurs in slice 11 and part of the chest wall is included in the parenchymal breast region. By contrast, with the “best” set of parameters (as found by repeating the algorithm and manually adjusting them), the lower row shows that the problem in slice 11 is corrected, with good matching of the pectoral muscle contour, but only at the cost of introducing an undersegmentation in slice 8, and, worse, losing the segmented breast region entirely in slice 6. In practice, where such problems occurred, it was necessary to edit the final segmentations manually. (Note on terminology: As shown in Fig. 6, the “BC‐FCM/heuristics (VaD)” method cannot reliably be run for the whole cohort using only default parameters and so we must describe the technique as semi‐ rather than fully automated. Even for cases where no manual editing or parameter adjustment need to be performed, human inspection is still required to confirm this. All subsequent cohort statistics will therefore use the nomenclature VsD to reflect this.)

We have run a similar analysis on all 16 cases for which we have duplicate manual segmentations by all three observers. The detailed results are shown in the Supplementary Information.

**Figure 7:**
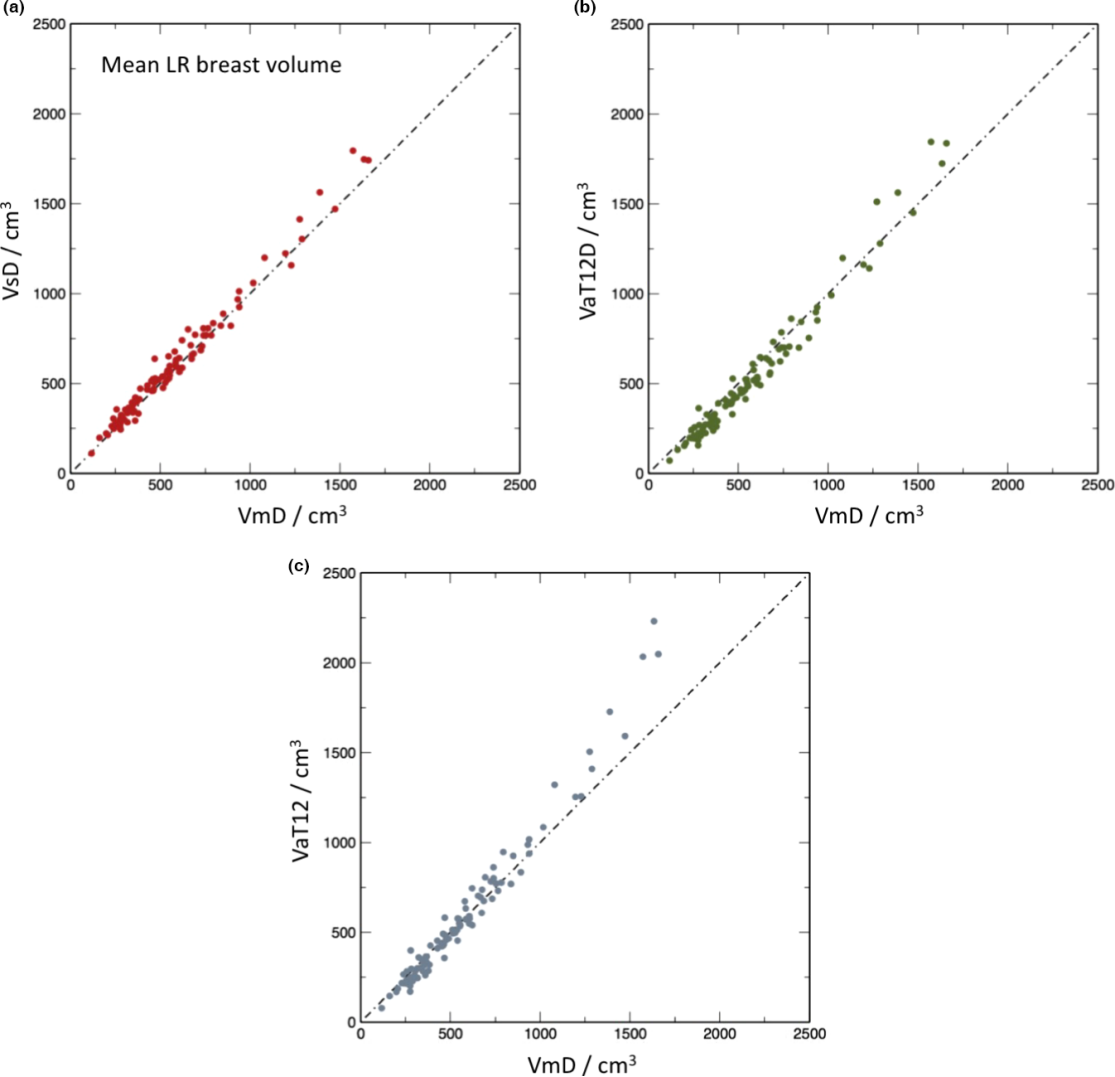
Scatter plots of mean left and right breast volumes in cm^3^ for the different methods in comparison to manual segmentation: (a) volume from semiautomatic segmentation of Dixon images (VsD) vs. volume from manual segmentation (VmD); (b) volume via automated segmentation from T_1_‐ and T_2_‐weighted images transformed to Dixon reference frame (VaT12FD) vs manual (VmD); (c) volume obtained from T_1_‐ and T_2_‐weighted images in native 3‐D reference frame (VaT12). [Color figure can be viewed at wileyonlinelibrary.com]

A second method of examining the relation between the volume segmentation results is to plot the total breast volume obtained by one method against that of another. In the scatter plots of Figs. 7(a)–7(c), the *x*‐ and *y*‐coordinates of each point represent the mean, for a single subject, of the left and right breast volumes evaluated, respectively, by the two methods under consideration. Figure 7(a) compares VsD, the semiautomated BC‐FCM method using Dixon image input, with the “gold‐standard” median manual segmentation, VmD, measured on the same Dixon dataset. Figure 7(b) gives results for the VaT12 method, which operates on the T1w and T2w datasets and evaluates the breast volume in the coordinate space of the T1w dataset. Finally, Fig. 7(c) looks at the effect of resampling the map generated by the algorithm in (b) with the spatial resolution and frame of reference of the Dixon data, which we term VaT12D. In each case, the line of identity is shown and Table IV reports the corresponding interclass correlations (ICC), representing the proportion of variance across participants shared between different ascertainment methods.

### 3.B. Fat–water segmentation

**Figure 8:**
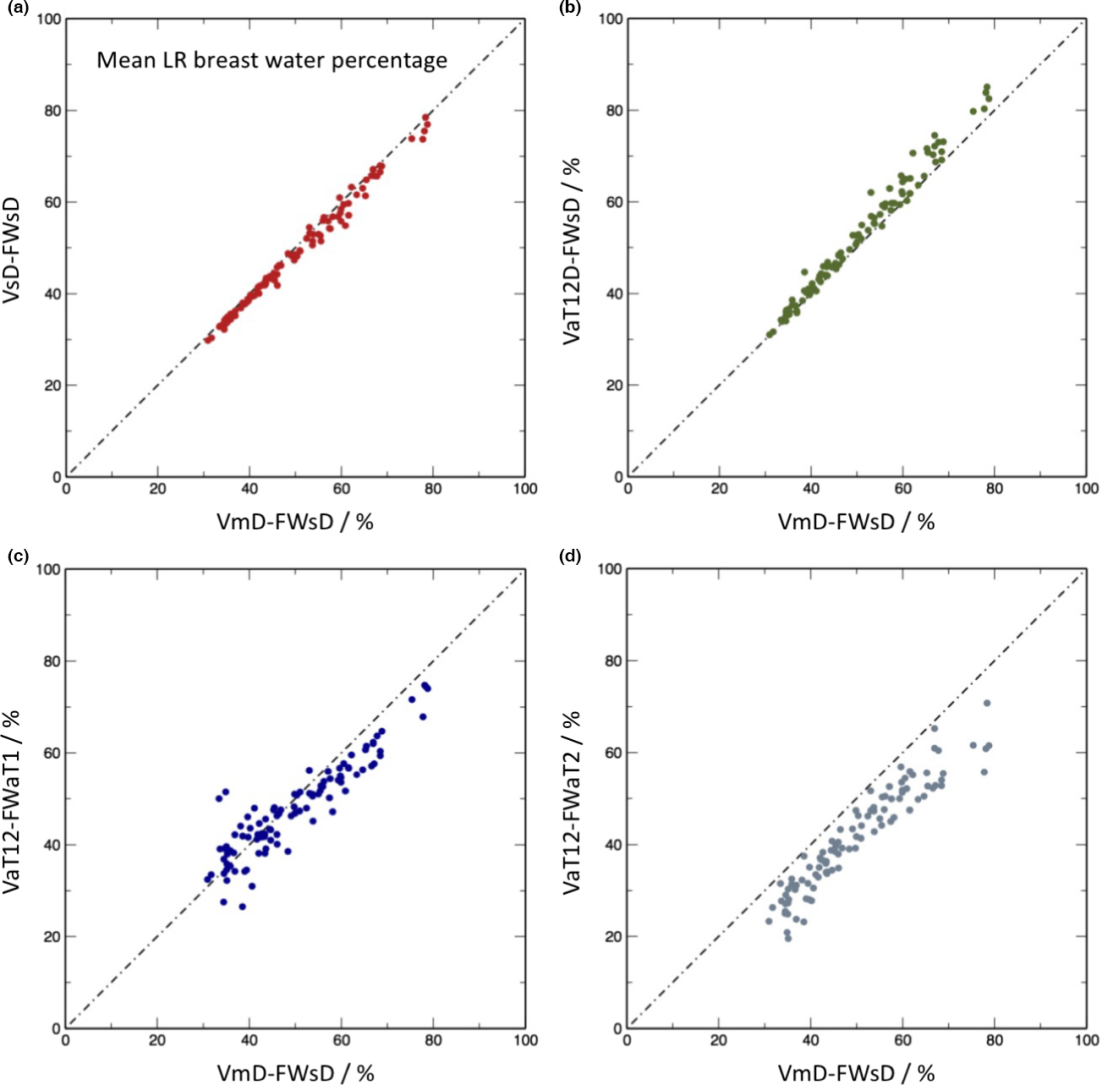
Scatter plots of mean left and right breast water percentage for the different methods in comparison with manual segmentation on Dixon images followed by percentage water estimation the using semiautomated Dixon image method: (a) semiautomatic segmentation of Dixon images followed by percentage estimate from Dixon image data (VsD‐FWsd); (b) volume via automated segmentation from T_1_‐ and T_2_‐weighted images transformed to Dixon reference frame (VaT12FD) followed by semiautomated percentage estimate from the Dixon data (VaT12D‐FWsd); (c) volume obtained from T_1_‐ and T_2_‐weighted images in native 3‐D reference frame, followed by automatic percentage estimate from T_1_‐weighted data (VaT12‐FWaT1); (d) as (c), but with the water percentage estimated from the T_2_‐weighted data. [Color figure can be viewed at wileyonlinelibrary.com]

**Figure 9:**
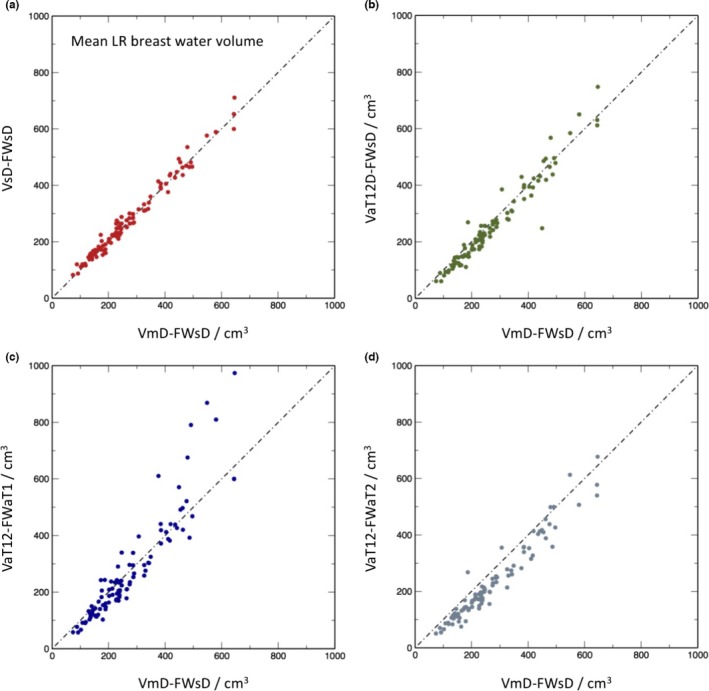
Scatter plots of mean left and right breast water volumes in cm^3^ for the different methods in comparison to VmD‐FWsD. For nomenclature see caption to Fig. 8. [Color figure can be viewed at wileyonlinelibrary.com]

Figures 8 and 9 present the results of the fat and water segmentation in the same format as for the total breast volume. In this case, however, a further option is available. Although the breast outline segmentation VaT12 requires both the T1w and T2w data, once this mask is available, it is possible to obtain two separate fat–water segmentations one using just the T1w and one using just the T2w data. These are denoted VaT12‐FWaT1 and VaT12‐FWaT2, respectively.

The interclass correlation (ICC) for total water volume, representing the proportion of variance across participants shared between the different ascertainment methods, are given in Table V.

### 3.C. Epidemiological results

**Figure 10:**
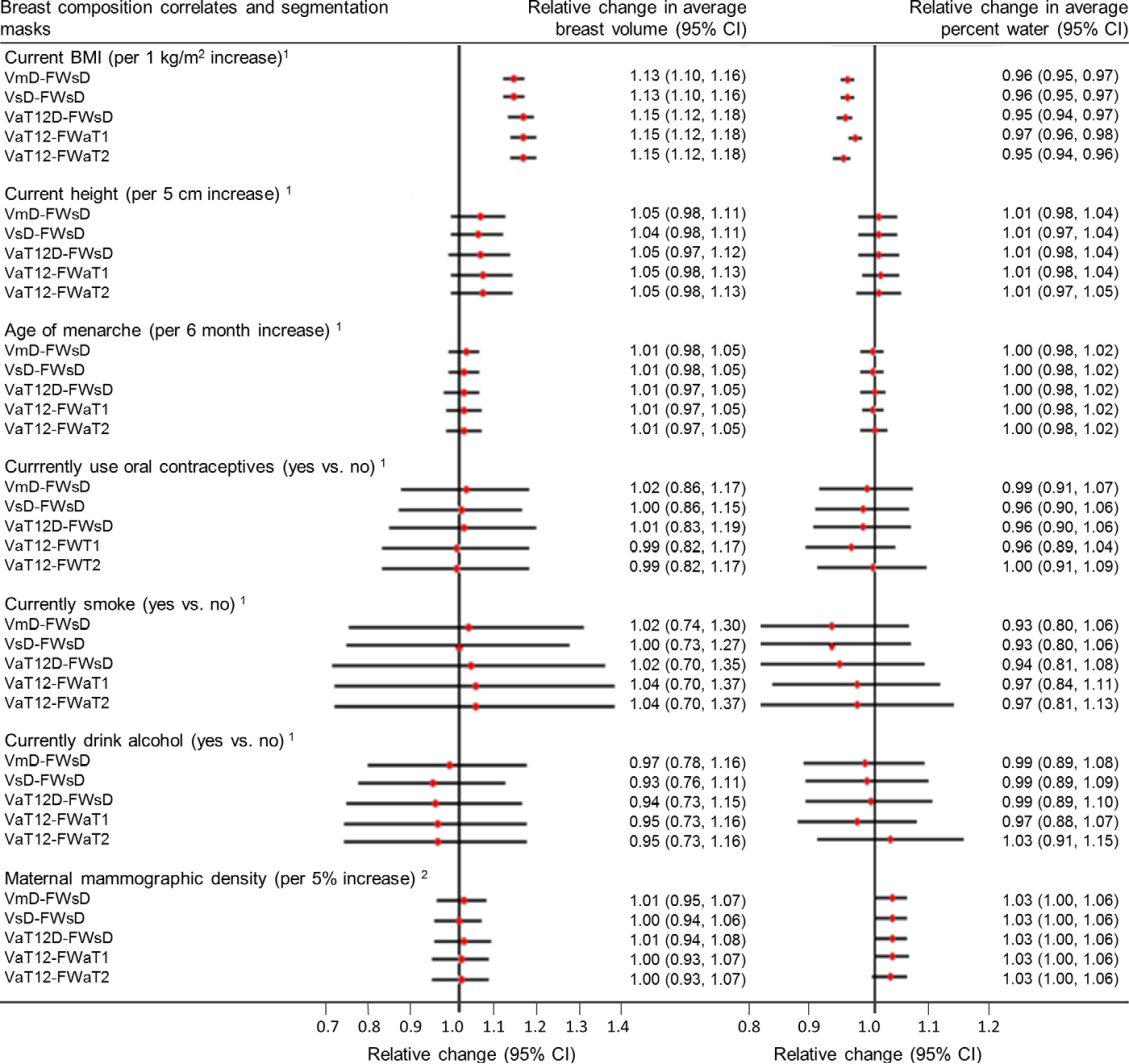
Results of epidemiological analysis. Relative change in geometric means of MR breast volume and percent water in relation to a unit increase, or category change, in each breast composition correlate variable. ^1^Models adjusted for current age in months and BMI at MR scan, where appropriate. ^2^Models restricted to young women for whom mammograms from their mothers could be retrieved (n = 33) adjusted for current age in months and BMI at MR scan and maternal age at mammogram and BMI in 2010 (median = 3y (IQR = 1.5y) prior to mammogram). For further details, see Supplementary Information. [Color figure can be viewed at wileyonlinelibrary.com]

A diagrammatic summary of the results of the epidemiological analysis is presented in Fig. 10 and further details of the work are reported as supplementary information.

Associations with both breast volume and breast water fraction were found for current body mass index (BMI). For a 1 kg m^−2^ increase in BMI, a relative change in breast volume of 1.13[1.10, 1.16] was observed for the cohort for both the VmD and VsD methods and the corresponding result for the VaT12 family of methods was 1.15[1.12, 1.18], where the figures in square brackets are the 95% confidence intervals. A smaller, but still important, decrease in breast water fraction was seen, and the corresponding statistics are VmD‐FWsD, VsD‐FWsD 0.96[0.95, 0.97], VaT12D‐FWsD 0.95[0.94, 0.97], VaT12‐FWaT1 0.97[096, 098], and VaT12‐FWT2 0.95[0.94, 0.96].

A weak association between current height and breast volume was also observed. For a 1 cm increase in height, the analysis methods gave the following relative increases in breast volume: VmD 1.05[0.98, 1.11], VsD 1.04[0.98,1.11], VaT12D‐FWsD was 1.05[0.97, 1.12], VaT12‐FWaT1 1.05[095, 1.03], and VaT12‐FWT2 1.05[0.95, 1.13]. However, height was not associated with breast water fraction.

No associations were found with any of age of menarche, use of oral contraception, smoking, alcohol intake or maternal mammographic density.

From the similarity of all these statistics, we conclude that the exact details of the segmentation methods are not significant at the level of this cohort analysis.

## 4 Discussion

Our results show that, as in many segmentation problems, the degree of success of the automated algorithms varies significantly between subjects. Figure 5 and Table 2 demonstrate excellent performance by all of the algorithms, whereas the degree of correspondence with the expert manual segmentation is considerably poorer in Fig. 6 and Table 3. However, it should be noted that even the expert human observer is less able to provide a good repeat segmentation.

**Table 3:** Dice and Jaccard coefficients for the difficult segmentation problem of Fig. 6

	Manual 1	Manual 2	BC‐FCM Orig	BC‐FCM /heuristics(best)	BC‐FCM Edited (VsD)	VaT12D
Dice coefficients						
Manual 1	1.000					
Manual 2	0.915	1.000				
BC‐FCM Orig	0.776	0.797	1.000			
BC‐FCM /heuristics(best)	0.836	0.792	0.782	1.000		
BC‐FCM Edited (VsD)	0.914	0.913	0.809	0.828	1.000	
VaT12D	0.796	0.771	0.728	0.818	0.795	1.000
Jaccard coefficients						
Manual 1	1.000					
Manual 2	0.843	1.000				
BC‐FCM Orig	0.634	0.662	1.000			
BC‐FCM /heuristics (best)	0.718	0.657	0.642	1.000		
BC‐FCM Edited (VsD)	0.842	0.840	0.679	0.707	1.000	
VaT12D	0.661	0.627	0.572	0.692	0.660	1.000

The ICCs for total breast volume in Table 4 demonstrate good agreement between all methods, but interestingly, slightly closer agreement between VaT12 and the two Dixon‐based methods (VmD or VsD) than between VaT12D and the Dixon methods. As described above, VaT12D is created by simply resampling VaT12 in the Dixon coordinate space, which has a coarser slice thickness, using appropriate blurring and nearest neighbor interpolation. Although movement between the Dixon and T1w or T2w scans could explain this disparity, registering the volumes did not improve the results. The resampling process appears to amplify the difference between VaT12 and VmD or VsD, but we have not analyzed this further, given that it is a relatively small effect.

**Table 4:** Interclass correlations for total breast volume segmentations

	VmD	VsD	VaT12D	VaT12
VmD	1.000			
VsD	0.990	1.000		
VaT12D	0.974	0.977	1.000	
VaT12	0.985	0.992	0.982	1.000

It would, of course, be interesting to compare the output of the VaT1T2 method directly with manual segmentation of the high‐resolution T1w dataset in its native reference frame, without the need to down‐sample. However, the workload involved in creating high‐resolution manual segmentations is prohibitive. In the Supplementary Information, we report anecdotal results for five such cases with full high‐resolution manual segmentations.

Also of note from comparison of the scatter‐plots of Fig. 7 is that each of methods VsD, VaT12D, and VaT12 increasingly overestimates the breast volume in comparison to VmD as the mean left and right breast size increases. This is most apparent for VaT12. The trend to larger error is, of course logical — similar percentage errors between the methods will result in greater absolute differences the larger the breast – but it is not currently clear why all methods are biased to *over*estimate the volume in this region. The method VaT12D also *under*estimates the breast volume for smaller breasts compared with the manual segmentation VmD and the reason for this, too, is unclear.

The biggest discrepancy between analysis methods, as shown by the scatter plots, is in the assessment of mean breast water volume (and, hence, water fraction — data not shown). The VsD‐FWsD and VaT12‐FWsD methods both use Dixon source data and differ from VmD‐FWsD only via the breast outline previously described. The methods all give very similar results (ICCs 0.995 and 0.992 in Table 5). By contrast, the correlation between the Dixon‐based VmD‐FWsD and VaT12‐FWaT1 is weaker, and the VaT12‐FWaT2 result additionally shows a bias (Fig. 8). However, it is important to note that the assumption that water fractions based on the Dixon method can be regarded as a gold standard for true parenchymal fraction is much less compelling than the previous assumption that VmD is the gold‐standard volume. We justify our choice of VmD‐FWsD as the method of comparison on the basis that it is consistent with previous work in the field^49^ (and indeed an improvement), but Ledger et al.^52^ have demonstrated that there is a significant degree of variability between different Dixon‐based methods, depending on the exact design of the pulse sequence. It is unsurprising that a segmentation based on a completely different MRI contrast mechanism should be less highly correlated. What is nevertheless highly encouraging is that the correlation remains as strong as it is — the worst value reported in Table 5 is 0.920 — and this suggests that the use of MRI as a modality will prove to be a robust choice for breast analysis.

**Table 5:** Interclass correlations for total water volume segmentations

	VmD‐FWsD	VsD‐FWsD	VaT12D‐FWsD	VaT12‐FWaT1	VaT12‐FWaT2
VmD‐FWsD	1.000				
VsD‐FWsD	0.995	1.000			
VaT12D‐FWsD	0.992	0.993	1.000		
VaT12‐FWaT1	0.920	0.921	0.924	1.000	
VaT12‐FWaT2	0.948	0.949	0.962	0.899	1.000

A salutary lesson from the scatter graphs is the constant need for vigilance and appropriate quality control when processing large cohorts of data. During the review of this paper, a referee noticed an outlier, which turned out to be the result of an easily corrected error that caused the mask for the entire right breast to be missing. Such “edge” cases, occurring very infrequently, remain a significant challenge in the adoption of automated pipelines. Any requirement for manual inspection of each dataset to check the output negates to some extent the advantages of fully automated segmentation processes, and an appropriate balance needs to be determined for each application.

Another feature highlighted by all of these results is the problem inherent in the use of quantitative metrics such as Dice and correlation coefficients, which (despite their apparent calculation “accuracy”) are a very blunt tool for analysing a complex situation. Are all of the voxels that fail to overlap equally important? Is much of the difference between the observer and the automated methods in fact caused by the choice of how much of the axilla is included and is this region of any significance biologically?

A first reading of the coefficients presented here suggests that the VsD breast outline segmentation, followed by the FWsD tissue segmentation method is the best‐performing of the computer‐aided tools presented here. But is it the most suitable? Ultimately, the choice of segmentation method needs to weigh up the following points:

- To what extent does the application demand a segmentation that is as good as that of an expert radiologist? Two extremes here might be the planning of radiotherapy treatment for an individual patient, where high correspondence is vital, and the calculation of epidemiological parameters for a Big Data cohort, where errors might well “average out.”
- To what extent is the ground truth knowable? For a given set of intra‐ and interobserver performance metrics evaluated on a test cohort, what performance thresholds should be regarded as “acceptable” for automated segmentations?
- How widely available are the required source data? As previously noted, the Dixon protocol is not routinely included in clinical examinations, thus limiting the applicability of breast density measurements based on the VsD‐FWsD method.
- How robust is the method?
- To what extent are speed, convenience and consistency of method to be preferred over accuracy?

In our case, consideration of all of the above led to the use of the VaT12 method, rather than VsD, for segmentation of the remaining 300 cases in the cohort (results not presented). This choice was made largely on the basis of improved automation and on the epidemiological evidence from the 200‐strong training and test datasets, as described in Section 3.C, where key epidemiological parameters were found to be identical, within confidence limits, for both methods.

## 5 Conclusion

We have presented what we believe to be the first detailed comparison on a large, population‐based cohort of two methods of breast‐outline segmentation based on completely different approaches. These have been coupled with two methods of fat–water discrimination based on fundamentally different MR contrast mechanisms. All combinations of the methods studied are in very strong agreement, as seen both visually and via interclass correlation coefficients, and are suitable for large‐scale epidemiological analysis. We have discussed the assumptions behind the methods and posed a number of general questions that we believe need to be answered each time a decision is made on whether and how to perform automated segmentation.

## Conflicts of interest

The authors are not aware of any conflicts of interest.

## Supporting information


**Appendix S1.** Data availability statement.
**Appendix S2.** Statistical and epidemiological analysis.
**Figure S1.** Exemplar MR images from a single subject, illustrating the different spatial resolution and contrast in the various image types acquired.
**Figure S2.** Concepts involved in the heuristic algorithms of the BC‐FCM refinement algorithm.
**Figure S3.** Distribution of breast volumes and percentage water as measured by the different segmentation and fat‐water estimation methods. Nomenclature for method names is as described in the main text.
**Figure S4.** Results of Bland‐Altman analysis of (A) breast volume measurements and (B) percentage water measurements obtained using different segmentation methods. Nomenclature of method names is as described in the main text.
**Table S1.** Dice and Jaccard coefficients obtained by comparing manual and automatically segmented masks.
**Table S2.** Dice and Jaccard coefficients obtained by comparing manual and automatically segmented masks for five representative cases in which the high‐resolution T1‐w datasets were fully manually segmented.
**Appendix S3.** MRI manual masking protocol.

## References

[1] McCormack VA , Silva IDS. Breast density and parenchymal patterns as markers of breast cancer risk: a meta‐analysis. Cancer Epidemiol Biomarkers Prev. 2006;15:1159–1169. 16775176 10.1158/1055-9965.EPI-06-0034

[2] Vilaprinyo E , Forne C , Carles M , et al. Cost‐effectiveness and harm‐benefit analyses of risk‐based screening strategies for breast cancer. Plos One. 2014;9:e86858. 10.1371/journal.pone.0086858 PMC3911927 24498285

[3] Price ER , Keedy AW , Gidwaney R , Sickles EA , Joe BN. The potential impact of risk‐based screening mammography in women 40‐49 years old. Am J Roentgenol. 2015;205:1360–1364. 26204111 10.2214/AJR.15.14668

[4] Ciatto S , Houssami N , Apruzzese A , et al. Categorizing breast mammographic density: intra‐ and interobserver reproducibility of bi‐rads density categories. Breast 2005;14:269–275. 16085233 10.1016/j.breast.2004.12.004

[5] Highnam R , Brady SM , Yaffe MJ , Karssemeijer N , Harvey J . Robust breast composition measurement ‐ volparatm In: Mart J, Oliver A, Freixenet J, Mart R, eds. Digital Mammography: 10th International Workshop, IWDM 2010, Girona, Catalonia, Spain, June 16–18, 2010. Proceedings. Berlin, Heidelberg: Springer Berlin Heidelberg; 2010: 342–349.

[6] Waade G , Highnam R , Hauge I , et al. Impact of errors in recorded compressed breast thickness measurement impacts on volumetric density classification using volpara v1.5.0 software. Med Phys. 2016;43:2870–2876. 27277035 10.1118/1.4948503

[7] Gubern‐Mérida A , Kallenberg M , Platel B , Mann RM , Mart R , Karssemeijer N . Volumetric breast density estimation from full‐field digital mammograms: a validation study. PLoS One. 2014;43:2870–2876. 10.1371/journal.pone.0085952 PMC3897574 24465808

[8] Thompson DJ , Leach MO , Kwan‐Lim G , et al. Assessing the usefulness of a novel MRI‐based breast density estimation algorithm in a cohort of women at high genetic risk of breast cancer: the UK MARIBS study. Breast Cancer Res. 2009;11:R80. 19903338 10.1186/bcr2447 PMC2815542

[9] Hayton P , Hayton P , Brady JM , et al. Analysis of dynamic MRbreast images using a model of contrast enhancement. Med Image Anal. 1997;1:207–24. 9873907 10.1016/s1361-8415(97)85011-6

[10] Twellmann T , Lichte O , Nattkemper TW. An adaptive tissue characterization network for model‐free visualization of dynamic contrast‐enhanced magnetic resonance image data. IEEE Trans Med Imaging. 2005;24:1256–1266. 16229413 10.1109/TMI.2005.854517

[11] Koenig M. Automatic cropping of breast regions for registration in MR mammography. Proc SPIE. 2005;5747:1563–1570.

[12] Yao J. Classification and calculation of breast fibroglandular tissue volume on SPGR fat suppressed MRI. Proc SPIE. 2005;5747:1942–1949.

[13] Lu WLW , Yao JYJ , Lu CLC , Prindiville S , Chow C . DCE‐MRI segmentation and motion correction based on active contour model and forward mapping. Seventh ACIS International Conference on Software Engineering, Artificial Intelligence, Networking, Parallel/Distributed Computing (SNPD’06); 2006:0–4.

[14] Giannini V , Vignati A , Morra L , et al. A fully automatic algorithm for segmentation of the breasts in DCE‐MR images In: Engineering in Medicine and Biology Society (EMBC), 2010 Annual International Conference of the IEEE. Buenos Aires: IEEE 3146–3149. 10.1109/IEMBS.2010.5627191 21096592

[15] Wang L , Platel B , Ivanovskaya T , Harz M , Hahn HK , Ieee . Fully automatic breast segmentation in 3d breast MRI. 2012 9th IEEE International Symposium on Biomedical Imaging (ISBI), 1024–1027; 2012.

[16] Wu S , Weinstein S , Kontos D. Atlas‐based probabilistic fibroglandular tissue segmentation in breast MRI. Med Image Comput Assist Interv 2012;15:437–45. 10.1007/978-3-642-33418-4_54 PMC4180245 23286078

[17] Wu S , Weinstein SP , Conant EF , Localio AR , Schnall MD , Kontos D . Fully automated chest wall line segmentation in breast MRI by using context information In: Proc. SPIE 8315, Medical Imaging 2012: Computer‐Aided Diagnosis, 2012 http://dx.doi.org/10.1117/12.911612

[18] Wu S , Weinstein SP , Conant EF , Kontos D . Automated fibroglandular tissue segmentation and volumetric density estimation in breast MRI using an atlas‐aided fuzzy c‐means method. Med Phys. 2013;12:122302. 10.1118/1.4829496 PMC3852242 24320533

[19] Wu S , Weinstein SP , Conant EF , Kontos D . Fully‐automated fibroglandular tissue segmentation and volumetric density estimation in breast MRI by integrating a continuous max‐flow model and a likelihood atlas In: SPIE Medical Imaging. Lake Buena Vista, FL: International Society for Optics and Photonics; 86701C.

[20] Gubern‐Merida A , Kallenberg M , Marti R , Karssemeijer N . Multi‐class probabilistic atlas‐based segmentation method in breast MRI In: Vitria J, Sanches JM, Hernandez M, eds. Pattern Recognition and Image Analysis, Ibpria 2011, Lecture Notes in Computer Science, Vol. 6669. Berlin, Heidelberg: Springer; 2011:660–667.

[21] Gubern‐Mérida A , Kallenberg M , Martí R , Karssemeijer N . Segmentation of the Pectoral Muscle in Breast MRI Using Atlas‐Based Approaches In: Ayache N, Delingette H, Golland P, Mori K. eds. Medical Image Computing and Computer‐Assisted Intervention ‐ MICCAI 2012. Lecture Notes in Computer Science, Vol. 7511. Berlin: Springer; 2012. 10.1007/978-3-642-33418-4_46 23286070

[22] Gubern‐Mérida A , Kallenberg M , Mann R , Marti R , Karssemeijer N. Breast segmentation and density estimation in breast MRI: a fully automatic framework. IEEE J Biomed Health Inform 2015;19:349–357. 25561456 10.1109/JBHI.2014.2311163

[23] Gallego‐Ortiz C , Martel A. Automatic atlas‐based segmentation of the breast in MRI for 3d breast volume computation. Med Phys. 2012;39:5835–5848. 23039622 10.1118/1.4748504

[24] Khalvati F , Gallego‐Ortiz C , Balasingham S , Martel AL. Automated segmentation of breast in 3‐d MR images using a robust atlas. IEEE Trans Med Imaging 2015;34:116–125. 25137725 10.1109/TMI.2014.2347703

[25] Gallego C , Martel AL . Automatic model‐based 3d segmentation of the breast in MRI Proc. SPIE 7962, Medical Imaging 2011: Image Processing, 796215. http://dx.doi.org/10.1117/12.877712.

[26] Ertas G , Gulcur HO , Tunaci M , Dursun M. k‐means based segmentation of breast region on MR mammograms. Magn Reson Mater Phys Biol Med 2006;19:317.

[27] Ertas G , Gulcur HO , Tunaci M , Osman O , Ucan ON. A preliminary study on computerized lesion localization in MRmammography using 3d nMITR maps, multilayer cellular neural networks, fuzzy c‐partitioning. Med Phys. 2008;35:195–205. 18293575 10.1118/1.2805477

[28] Wang C‐M , Mai X‐X , Lin G‐C , Kuo C‐T. Classification for breast MRI using support vector machine. 8th IEEE International Conference on Computer and Information Technology Workshops: Cit Workshops 2008, Proceedings, 362–367;2008.

[29] Wang Y , Morrell G , Heibrun ME , Payne A , Parker DL. 3d multi‐parametric breast MRI segmentation using hierarchical support vector machine with coil sensitivity correction. Acad Radiol. 2013;20:137–147. 23099241 10.1016/j.acra.2012.08.016 PMC3567300

[30] Klifa C . Quantification of breast tissue index from MR data using fuzzy clustering Engineering in Medicine and Biology Society, 2004. IEMBS ‘04. 26th Annual International Conference of the IEEE. San Francisco: IEEE; 2004. 10.1109/IEMBS.2004.1403503 17272023

[31] Klifa C , Carballido‐Gamio J , Wilmes L , et al. Magnetic resonance imaging for secondary assessment of breast density in a high‐risk cohort. Magn Reson Imaging. 2010;28:8–15. 19631485 10.1016/j.mri.2009.05.040 PMC4087111

[32] Yang S‐C , Wang C‐M , Hsu H‐H , et al. Contrast enhancement and tissues classification of breast MRI using Kalman filter‐based linear mixing method. Comput Med Imaging Graph 2009;33:187–196. 19135862 10.1016/j.compmedimag.2008.12.001

[33] Nie K , Chen J‐H , Chan S , et al. Development of a quantitative method for analysis of breast density based on three‐dimensional breast MRI. Med Phys. 2008;35:5253–5262. 19175084 10.1118/1.3002306 PMC2673600

[34] Sathya A , Senthil S , Samuel A. Segmentation of breast MRI using effective fuzzy c‐means method based on support vector machine. Proceedings of the 2012 World Congress on Information and Communication Technologies, 67–72; 2012.

[35] Lin M , Chan S , Chen J‐H , et al. A new bias field correction method combining N3 and FCM for improved segmentation of breast density on MRI. Med Phys. 2011;38:5–14. 21361169 10.1118/1.3519869 PMC3017578

[36] Lin M , Chen J‐H , Wang X , Chan S , Chen S , Su M‐Y. Template‐based automatic breast segmentation on MRI by excluding the chest region. Med Phys. 2013;40:122301‐1–122301‐10. 24320532 10.1118/1.4828837 PMC3843758

[37] Ertas G , Doran SJ , Leach MO. A computerized volumetric segmentation method applicable to multicentre MRI data to support computer aided breast tissue analysis, density assessment and lesion localization. Med Biol Eng Comput. 2017;55:57–68. 27106750 10.1007/s11517-016-1484-y PMC5222930

[38] Dixon WT. Simple proton spectroscopic imaging. Radiol. 1984;153:189–194. 10.1148/radiology.153.1.6089263 6089263

[39] England PH . Breast screening: professional guidance; 2016.

[40] Boyd A , Golding J , Macleod J , et al. Cohort profile: the ’children of the 90s’ – the index offspring of the avon longitudinal study of parents and children. Int J Epidemiol. 2013;42:111–127. 22507743 10.1093/ije/dys064 PMC3600618

[41] ALSPAC . http://www.bris.ac.uk/alspac/researchers/data-access/data-dictionary; Accessed May 31, 2017.

[42] Pham DL , Prince JL. An adaptive fuzzy c‐means algorithm for image segmentation in the presence of intensity inhomogeneities. Pattern Recognit Lett. 1999;20:57–68.

[43] Tustison NJ , Avants BB , Cook PA , et al. N4itk: Improved n3 bias correction. IEEE Trans Med Imaging. 2010;29:1310–1320. 20378467 10.1109/TMI.2010.2046908 PMC3071855

[44] National Library of Medicine . Insight segmentation and registration toolkit (itk), http://www.itk.org/. Accessed May 31, 2017.

[45] Gubern‐Merida A , Kallenberg M , Marti R , Karssemeijer N. Segmentation of the pectoral muscle in breast MRI using atlas‐based approaches. Med Image Comput Assist Interv. 2012;15:371–8. 10.1007/978-3-642-33418-4_46 23286070

[46] Wang L , Filippatos K , Friman O , Hahn HK . Fully automated segmentation of the pectoralis muscle boundary in breast MR images Proc. SPIE 7963, Medical Imaging 2011: Computer‐Aided Diagnosis, 796309. http://dx.doi.org/10.1117/12.877645.

[47] Gubern‐Mérida A , Wang L , Kallenberg M , Marti R , Hahn HK , Karssemeijer N . Breast segmentation in MRI: quantitative evaluation of three methods. Medical Imaging 2013: Image Processing 8669; 2013.

[48] Griffn LD. The second order local‐image‐structure solid. IEEE Trans Pattern Anal Mach Intell. 2007;29:1355–1366. 17568140 10.1109/TPAMI.2007.1066

[49] Poon CS , Bronskill MJ , Henkelman RM , Boyd NF. Quantitative magnetic resonance imaging parameters and their relationship to mammographic pattern. J Natl Cancer Inst. 1992;84:777–781. 1573664 10.1093/jnci/84.10.777

[50] Song H , Cui X , Sun F. Breast tissue 3d segmentation and visualization on MRI. Int J Biomed Imaging. 2013;2013:859746. 23983676 10.1155/2013/859746 PMC3745868

[51] Van Leemput K , Maes F , Vandermeulen D , Suetens P. Automated model‐based tissue classification of MR images of the brain. IEEE Trans Med Imaging. 1999;18:897–908. 10628949 10.1109/42.811270

[52] Ledger AEW , Scurr ED , Hughes J , et al. Comparison of dixon sequences for estimation of percent breast fibroglandular tissue. PLoS One. 2016;11:e0152152. 27011312 10.1371/journal.pone.0152152 PMC4806997

