# Supplementary material for: Breast MRI segmentation for density estimation: Do different methods give the same results and how much do differences matter?
Source: Med Phys. 2017 Jul 25;44(9):4573–92. doi: 10.1002/mp.12320 (PMC5697622; doi:10.1002/mp.12320)

**Supporting information**

**Appendix S1: Data availability statement**

There are ethical restrictions to the availability of the data. ALSPAC is run as a resource to be used by the research community. The study website describes the resouce and summarises the data available, including the Data Dictionary which is available to download as a zipped PDF file here: <http://www.bristol.ac.uk/alspac/researchers/data-access/data-dictionary/>.

To access the data researchers are required to complete a proposal, which is reviewed by the Executive. Research proposals can be submitted online (https://proposals.epi.bristol.ac.uk/), or the Executive can be contacted here, [alspac-exec@bristol.ac.uk](mailto:alspac-exec@bristol.ac.uk)**.**

**Appendix S2: Statistical and epidemiological analysis**

The distributions of breast measurements obtained from the different segmentation methods in the validation set (n=100) were compared. Inter-class correlation (ICC) for each breast measure, representing the proportion of variance across participants shared between the different ascertainment methods, were estimated: the higher the ICC, the more similar the estimates across methods.

Bland-Altman analysis was used to assess agreement of the breast segmentation (total breast volume), and the internal segmentation of water and fat volumes (calculated using the water/fat fraction, respectively, and total breast volume), and percent water obtained from Dixon, T_1_-w and T_2_‑w images via manual, semi-automated and automated methods. The mean difference in breast measure estimates and limits of agreement (LOA, calculated as mean difference ± 1.96 standard deviation of the difference and representing 95% confidence intervals of the difference) between types of MR images and segmentation methods were calculated. Linear regression models were fitted to test if the mean difference in breast measure estimates was associated with the average value (i.e. the average of the values yielded by the two methods being compared). For breast volume and fat-water segmentation comparisons, the VmD and VmD-FWsD methods, respectively, were used as reference.

Appropriate linear and logistic regression models were used to examine associations of average total breast, fat and water volumes, and percent water, as measured using different MR images and segmentation methods, with selected established and potential mammographic density correlates. Breast measures were log-transformed and the exponentiated estimated regression parameters represent the relative change (RC) in breast measure with a unit increase, or category change, in the exposure of interest (with 95% confidence intervals (95% CI) calculated by exponentiating the original 95% CIs). Age at menarche (months), height (cm) and BMI (height (cm)/ weight (kg)2) at MR were treated as continuous variables and centred at the mean. Current hormone contraceptive use, cigarette smoking and alcohol drinking were treated as binary (yes/no) variables. Mother’s mammographic percent density (%) was averaged between both breasts, and maternal age (months) at mammography and clinically measured or self-reported maternal BMI (median 3 years (inter-quartile range (IQR) = 1.5 years) prior to mammography)) were used as continuous measures and centred at the mean. Variables were included as potential determinants of breast measures, or as confounding factors, where appropriate.

Data analysis was conducted with STATA statistical software, version 14. All P values presented are from two-sided tests of statistical significance.

**Figure S1: Exemplar MR images from a single subject, illustrating the different spatial resolution and contrast in the various image types acquired**


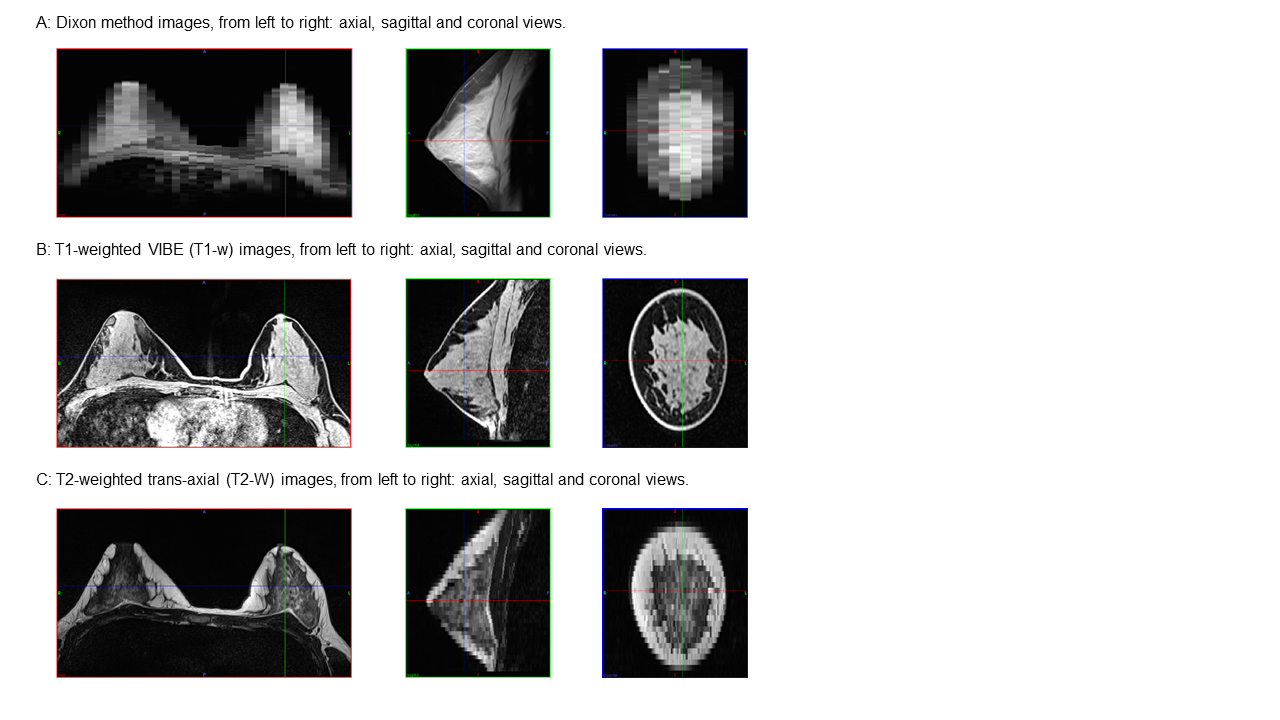


**Figure S2: Concepts involved in the heuristic algorithms of the BC-FCM refinement algorithm**

**
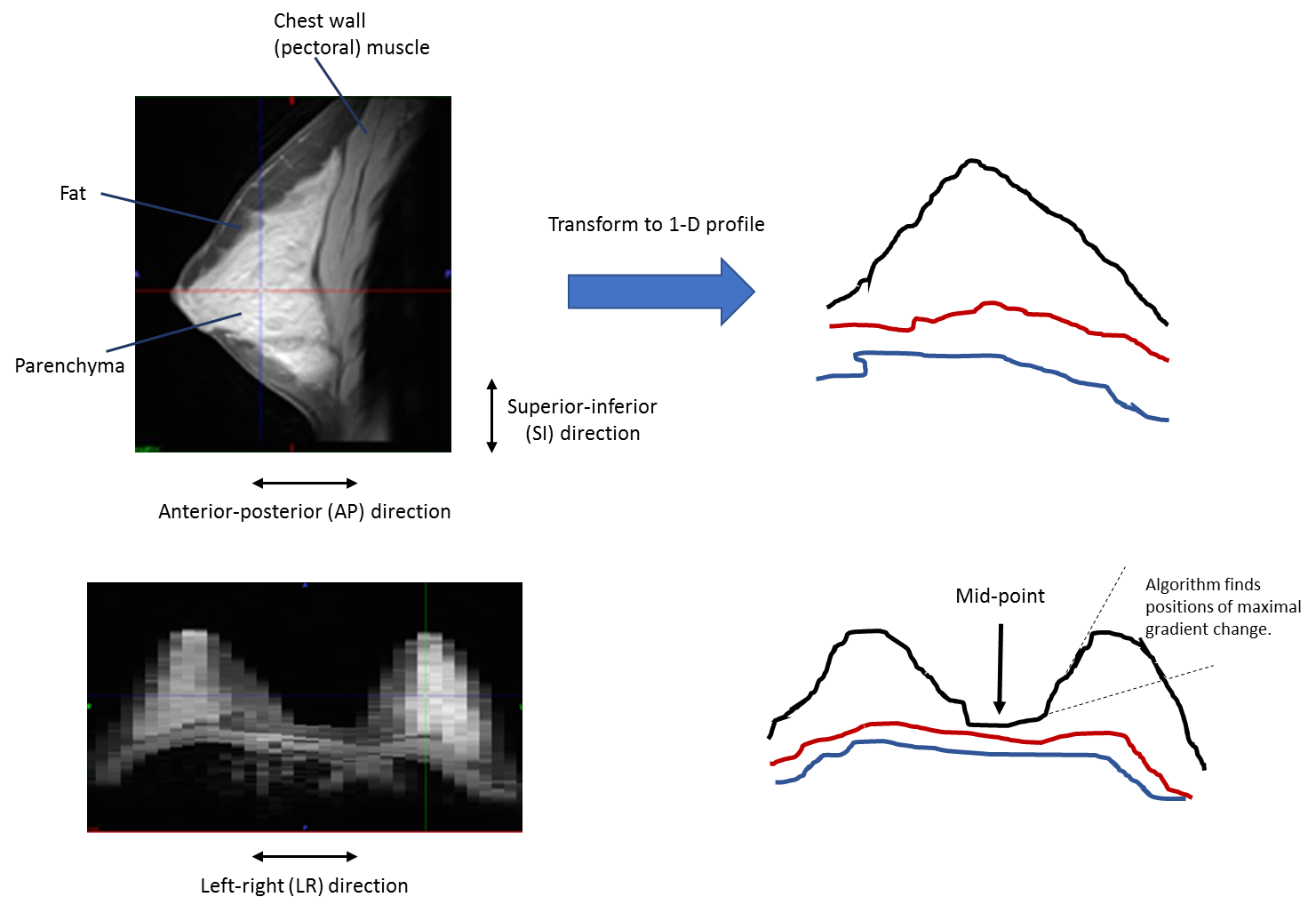
**

**Figure S3: Distribution of breast volumes and percentage water as measured by the different segmentation and fat-water estimation methods. Nomenclature for method names is as described in the main text.**


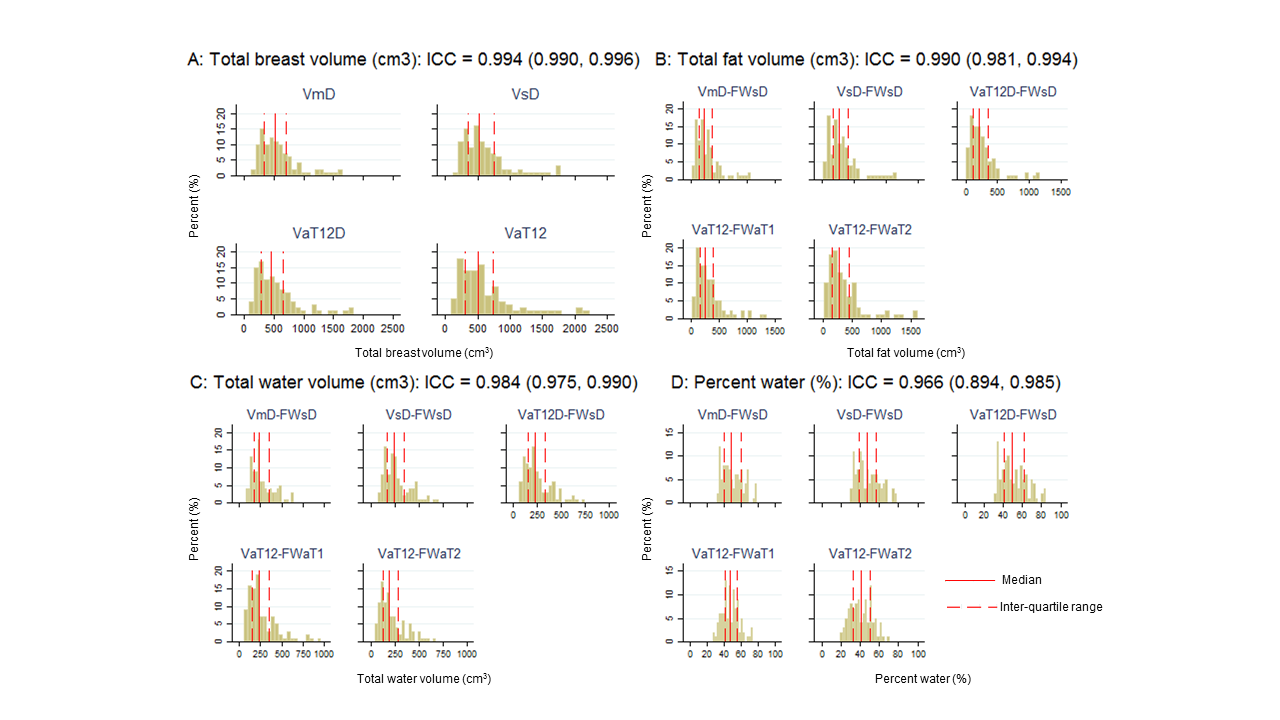


**Figure S4: Results of Bland-Altman analysis of (A) breast volume measurements and (B) percentage water measurements obtained using different segmentation methods. Nomenclature of method names is as described in the main text.**


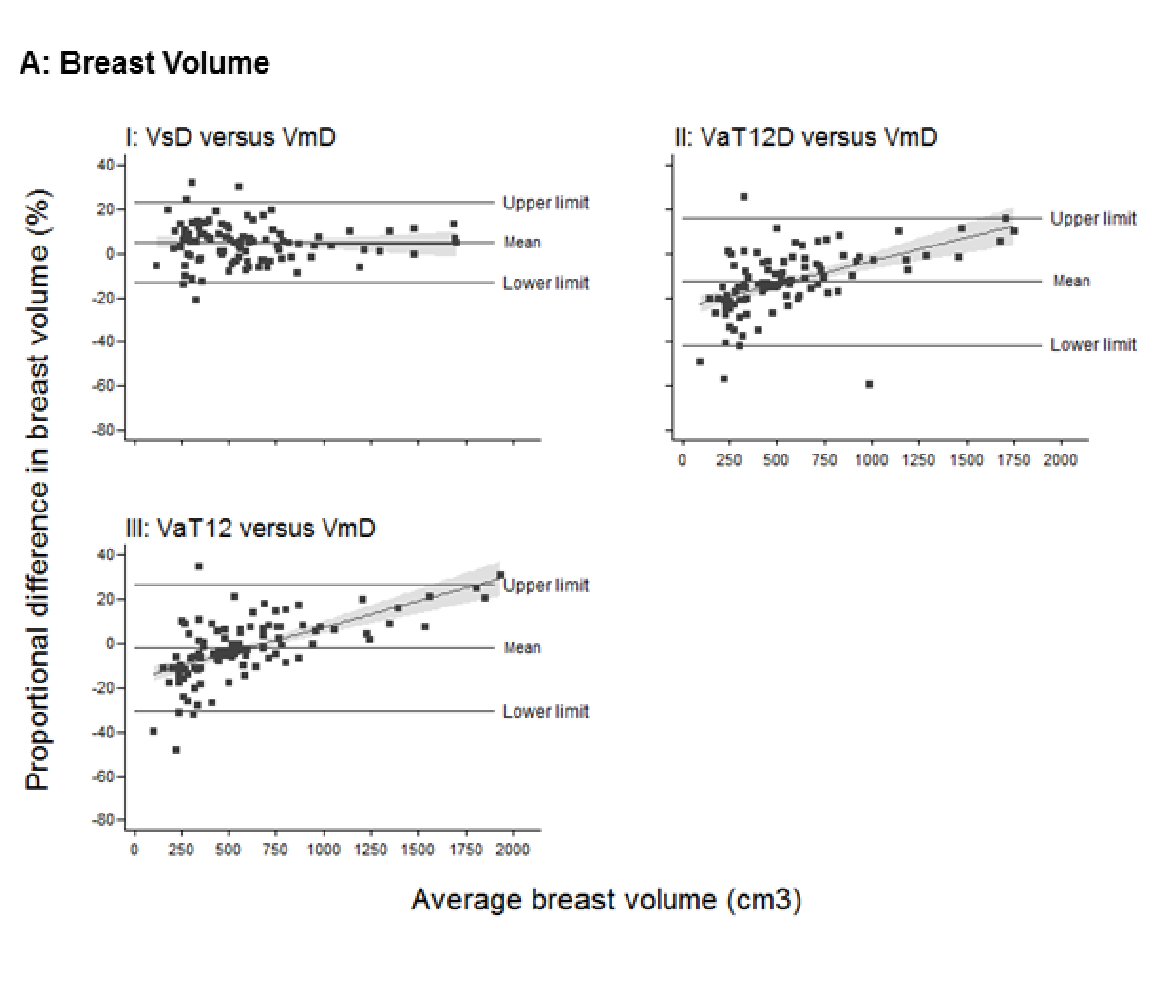


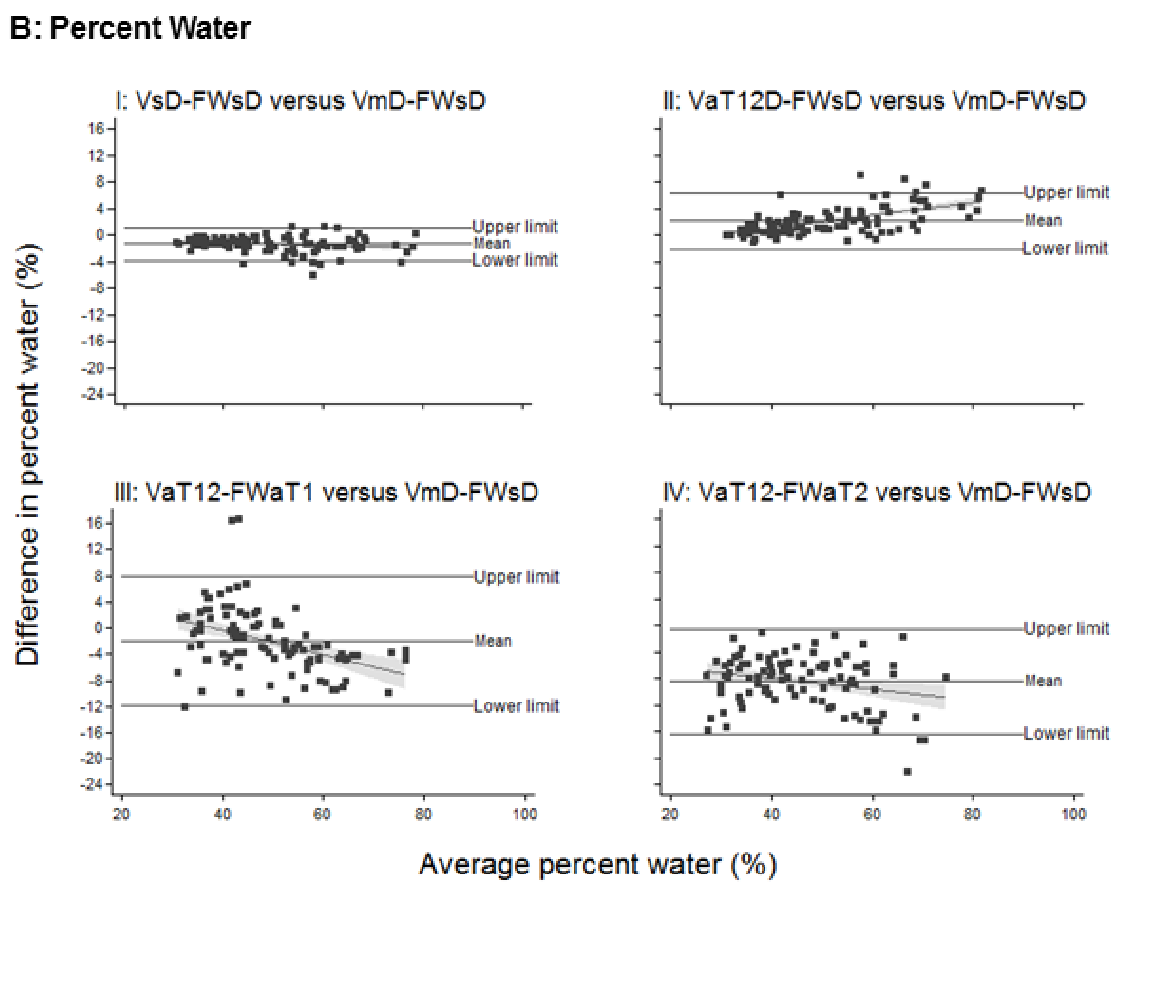


**Table S1: Dice and Jaccard coefficients obtained by comparing manual and automatically segmented masks**

Here we report on the results for 16 subjects for whom manual segmentations were performed by all three observers. All manual segmentations were drawn on the Dixon image data and the same operation was performed twice by each observer to give two independent versions of each mask.

Results are presented in the format:

*median* [*lower quartile, upper quartile*]

where the statistics are calculated over the 16 subjects using the IDL (Harris Geospatial Solutions) function createboxplotdata.

“BC-FCM original” refers to the algorithm created for the evaluation of the training cohort data. Insight from that first segmentation trial and initial comparison with manual results led to a modified BC-FCM algorithm using additional heuristics, as described in the main text. This is described here as “BC-FCM heuristics”, matching the notation of Table II, and this was run on the same set of cases.

The main findings are:

• The median intra-observer Dice coefficients for the three observers were 0.943, 0.922 and 0.906.

• The median inter-observer Dice coefficients varied from 0.898 to 0.936.

• The median Dice coefficients for the 16 subjects, calculated between the first version of the BC-FCM segmentation (VsD) and the different manual segmentations, varied between 0.822 and 0.851.

• After modification using knowledge gained from the training dataset, the revised BC-FCM algorithm (VsD) led to corresponding median values ranging from 0.865 to 0.885. The results showed an improvement in 12/16 cases, virtually no change in two cases, and a major degradation in the other two cases, suggesting that further algorithm “tweaking” may be necessary.

• The VaT12D masks gave median Dice coefficients ranging from 0.812 to 0.864.


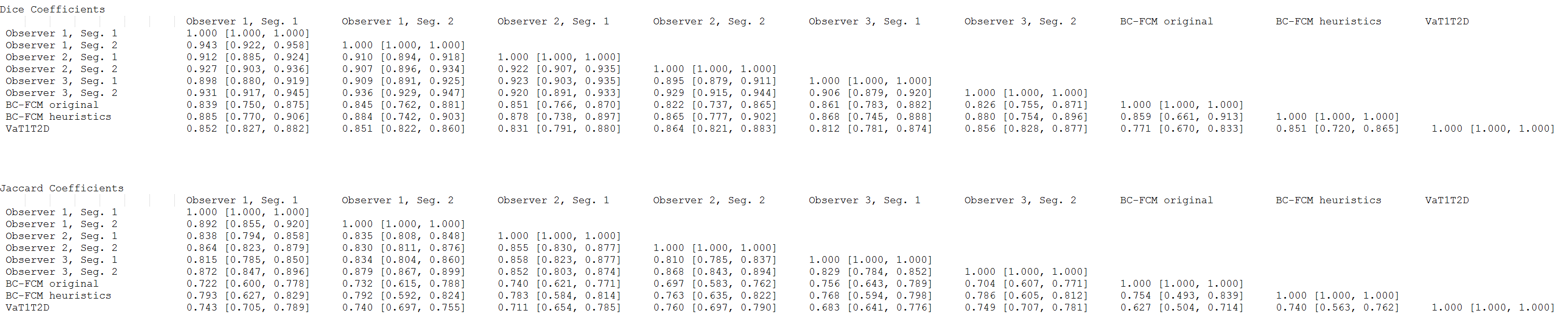


**Table S2: Dice and Jaccard coefficients obtained by comparing manual and automatically segmented masks for five representative cases in which the high-resolution T_1_-w datasets were fully manually segmented**

**
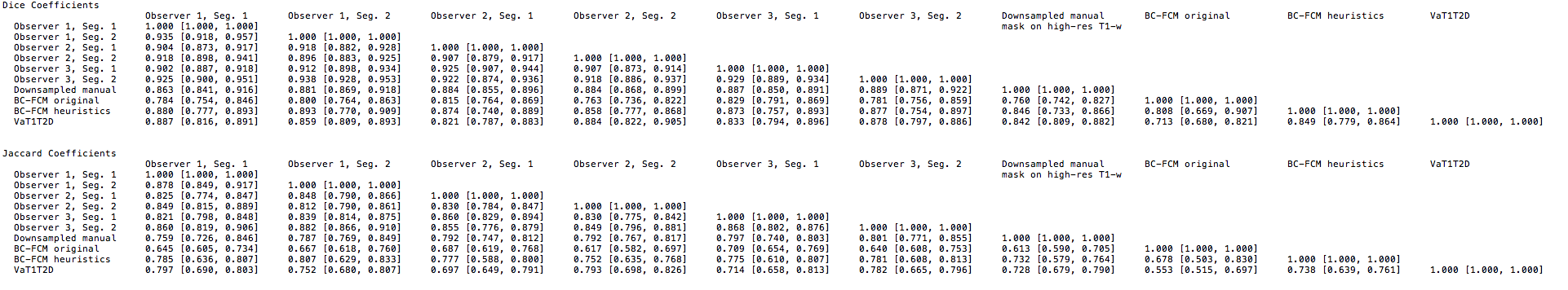
**

We report anecdotal evidence for five subjects drawn from the set described by Table S1.

The manual and automatic segmentations described in the main text are in the frame of reference of the Dixon image data. These are compared here with manual segmentations by Observer 1 on all slices of the high-resolution T_1_-w dataset.

Results are presented in the format:

*median* [*lower quartile, upper quartile*]

where the statistics are calculated over the five subjects using the IDL (Harris Geospatial Solutions) function createboxplotdata

and the other notation is as in Table S1.

For these five subjects, we also calculated the total breast volume from each mask and normalised it to the volume obtained using the first manual mask created by Observer 1. From these normalised breast volume measurements, we calculated the same summary statistics:

*median* [*lower quartile, upper quartile*]

and the results are as follows:

Observer 1, Seg. 1         1.000 [1.000, 1.000]

Observer 1, Seg. 2         1.046 [0.984, 1.103]

Observer 2, Seg. 1         1.097 [1.091, 1.188]

Observer 2, Seg. 2         0.951 [0.926, 1.032]

Observer 3, Seg. 1         1.134 [1.060, 1.163]

Observer 3, Seg. 2         1.055 [0.985, 1.129]

Downsampled manual         1.018 [0.979, 1.064]

High-res. T1-w mask        1.140 [1.069, 1.171]

BC-FCM Original 1.323 [1.294, 1.533]

BC-FCM Heuristics 1.057 [0.729, 1.090]

VaT1T2D 0.850 [0.769, 0.970]

The statistics are obtained from a very small (potentially unrepresentative) set of subjects and so it is difficult to draw any firm conclusions. However, it is noticeable that the volumes obtained from the “down-sampled” VaT1T2D method are, in general, lower than the manual segmentation on the Dixon dataset. Conversely, the volumes from the native high-resolution manual segmentation are slightly higher. Nevertheless, the volumes segmented on the high-resolution dataset agree with those obtained by the same observer on the Dixon data to a much better degree than might be expected from the poor agreement in Dice coefficient.

**Appendix S3: MRI manual masking protocol**

Using ITK-SNAP – open DICOM Image series and use volume sag_dixon_bilateral_in

When opening the MRI DICOM images, first set up the contrast-level of the images (tools > image contrast > contrast adjustment) and zoom to fit.

The mask should follow the contour of the breast.

The top of the breast should be cut close to where the contour flattens meets the chest. The cut should be a horizontal line.


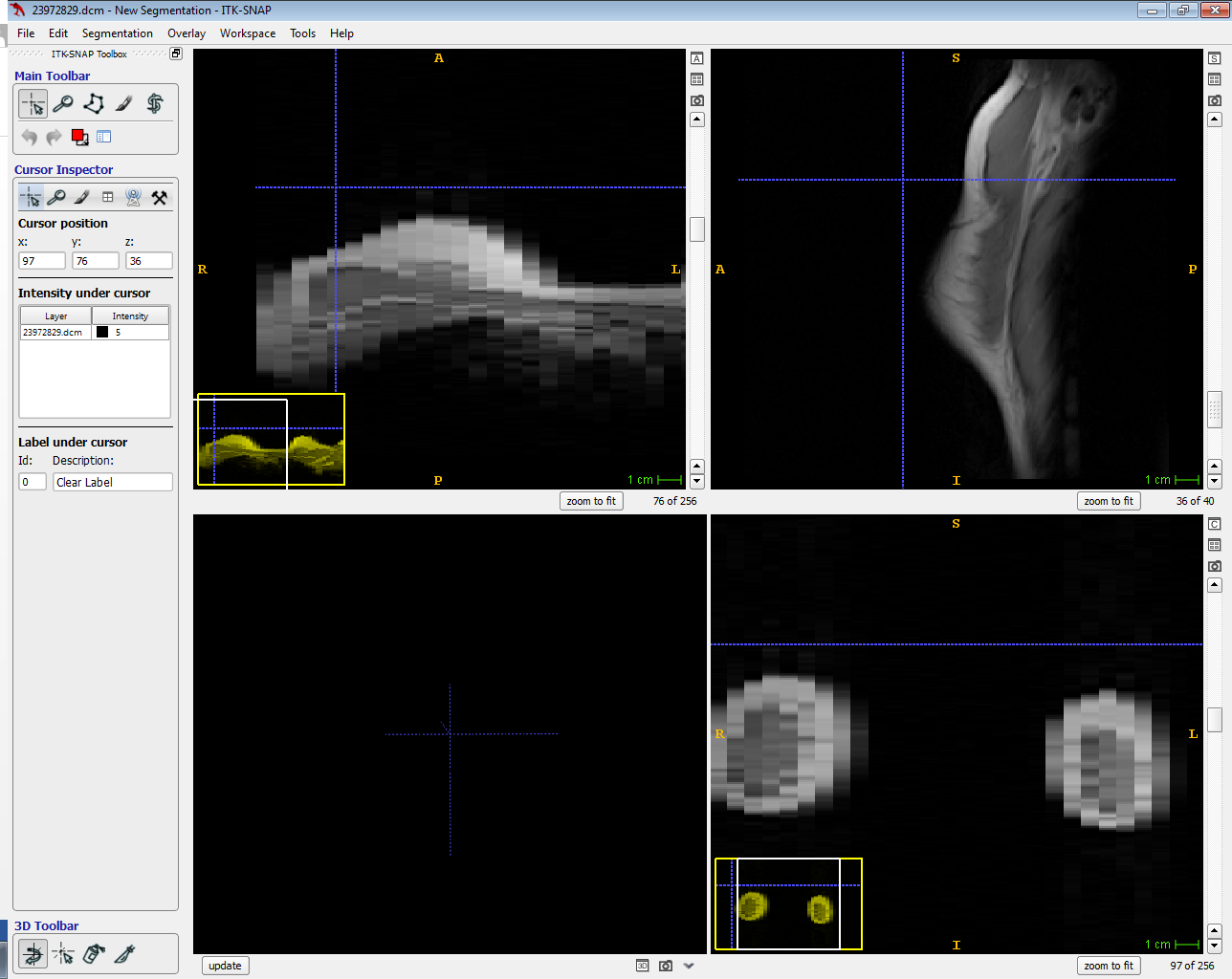

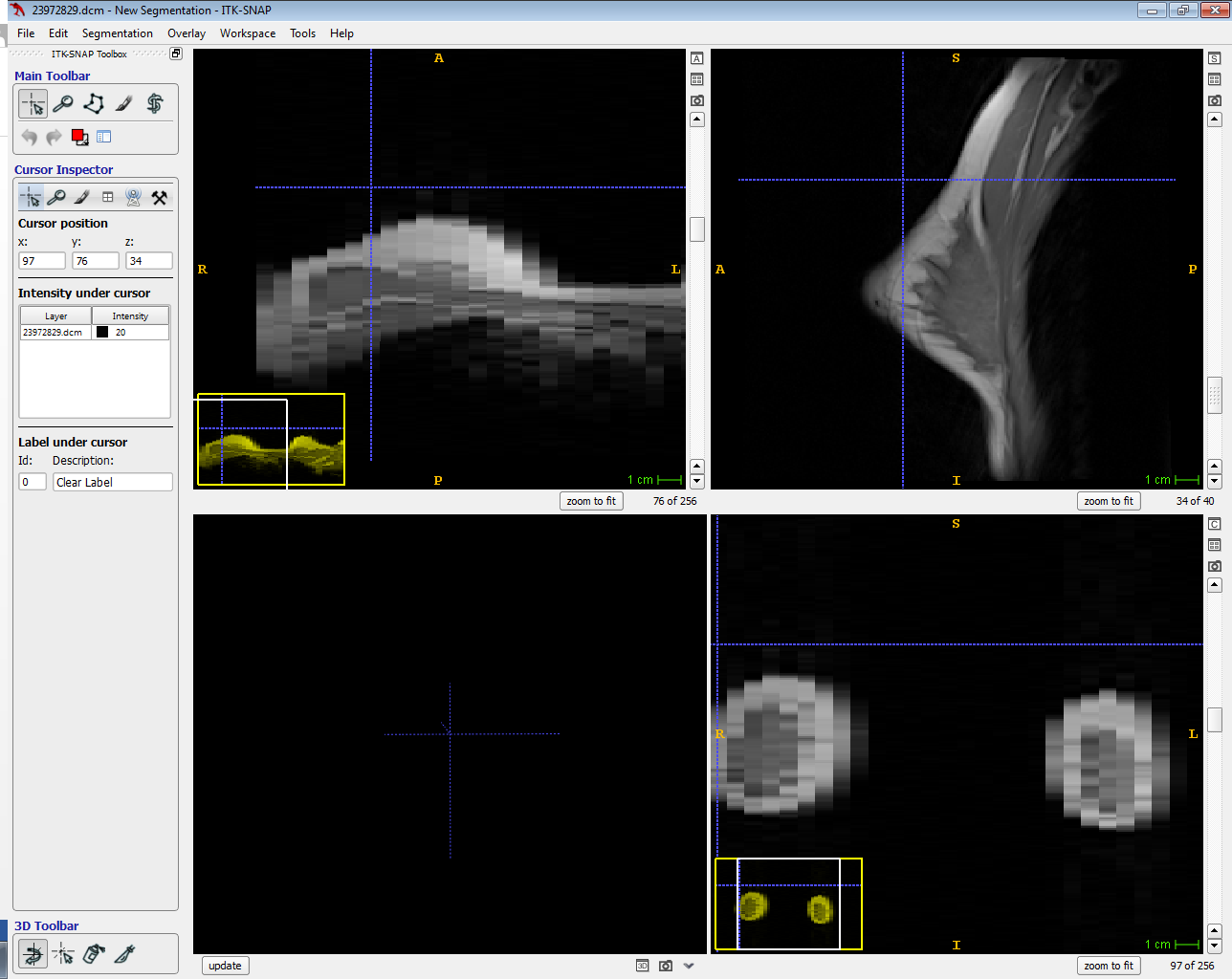


The bottom of the breast should be cut where the breast meets the chest wall, and could be either a horizontal or diagonal line, depending on the shape of the breast.


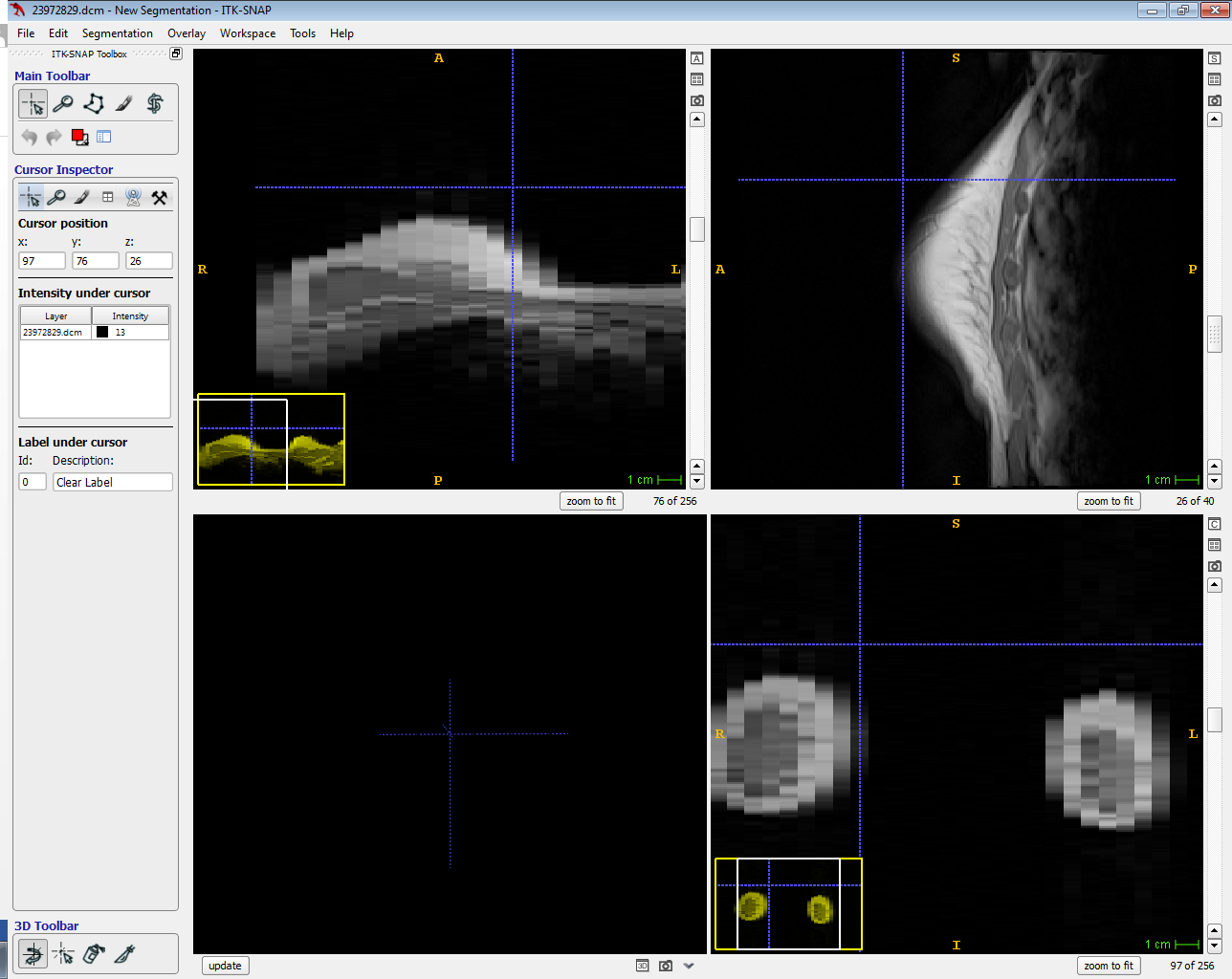

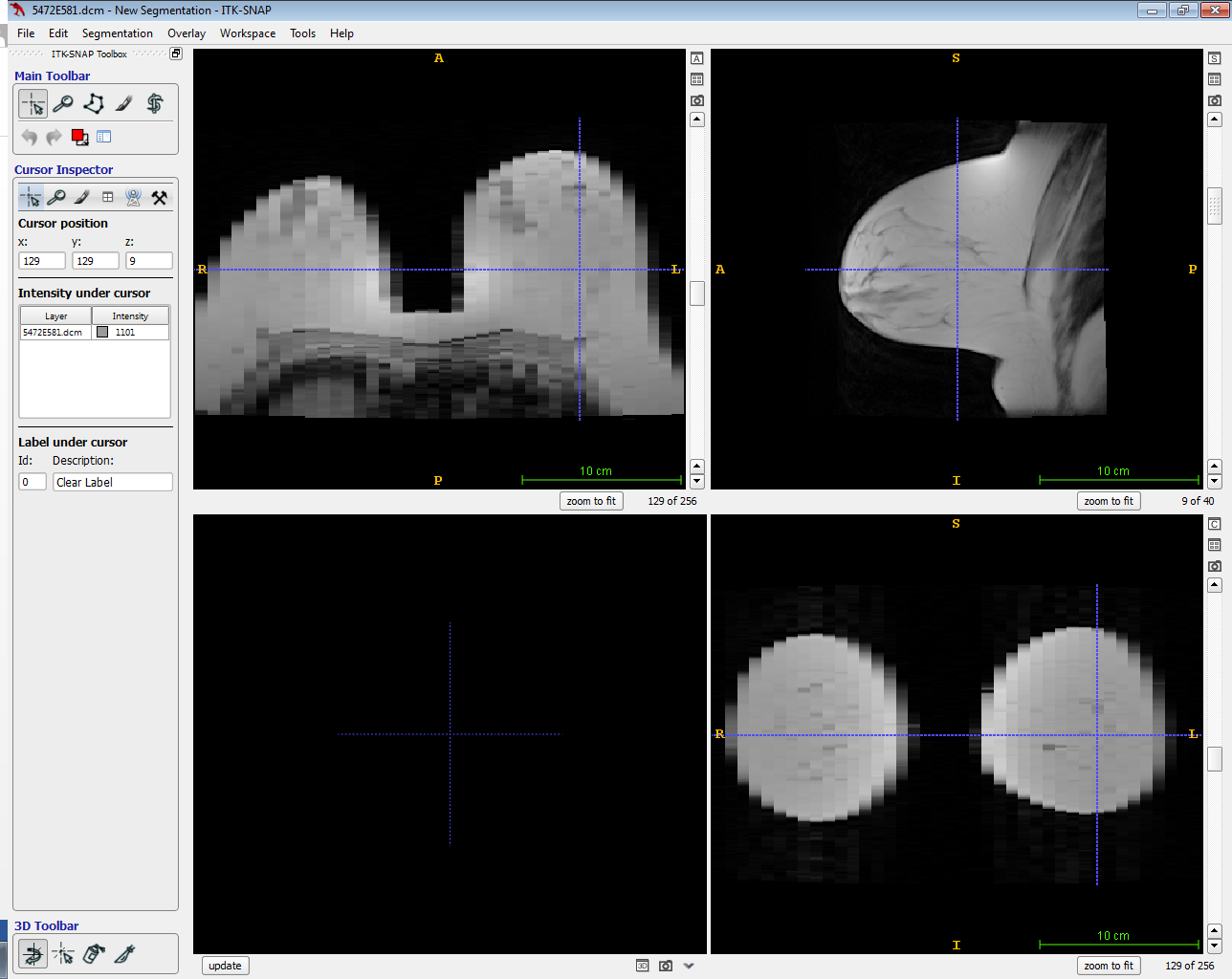


To ensure consistency within individuals, bring the mask forward from the previous image and use as a guide for defining the top and bottom of the breast in the next image.


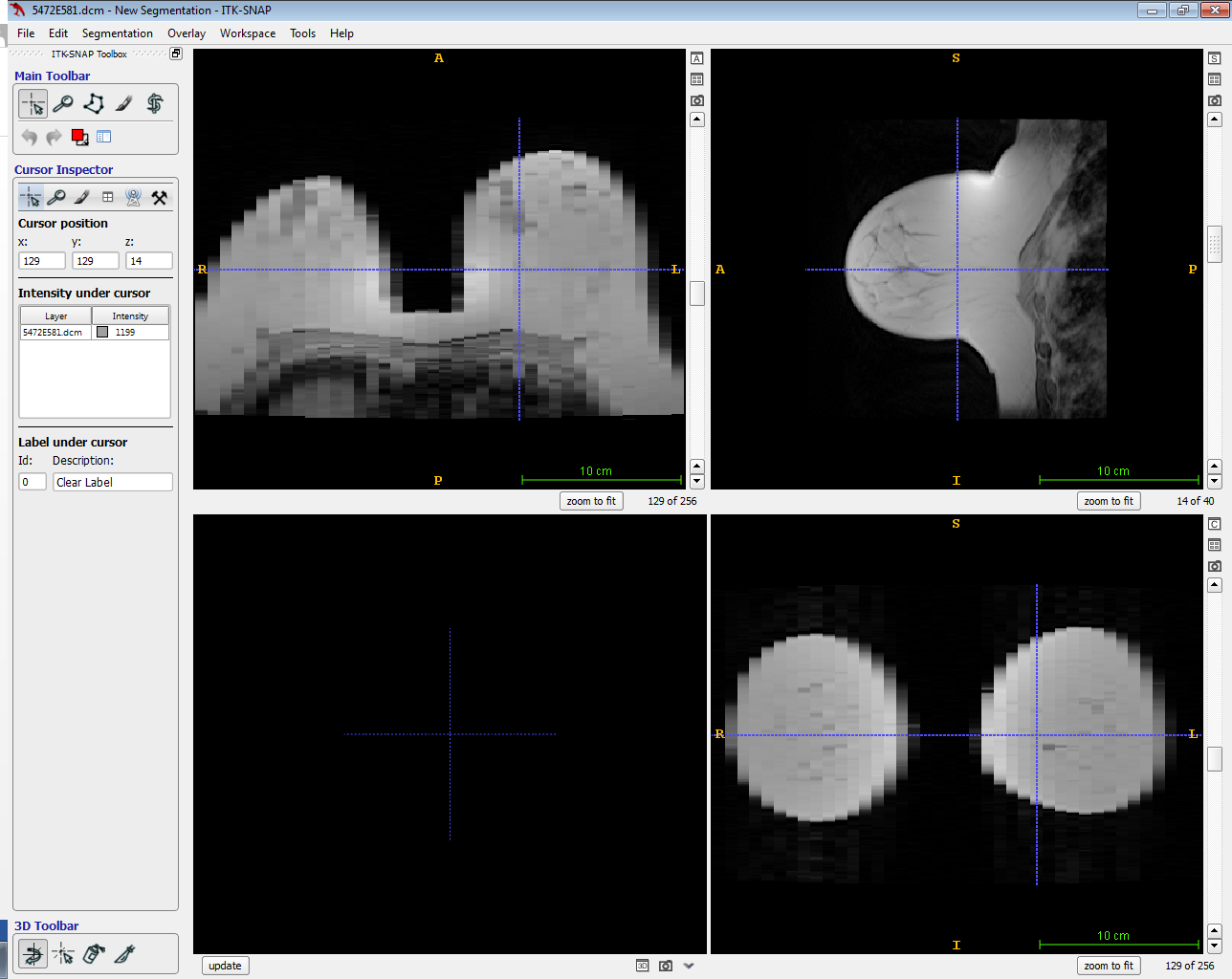

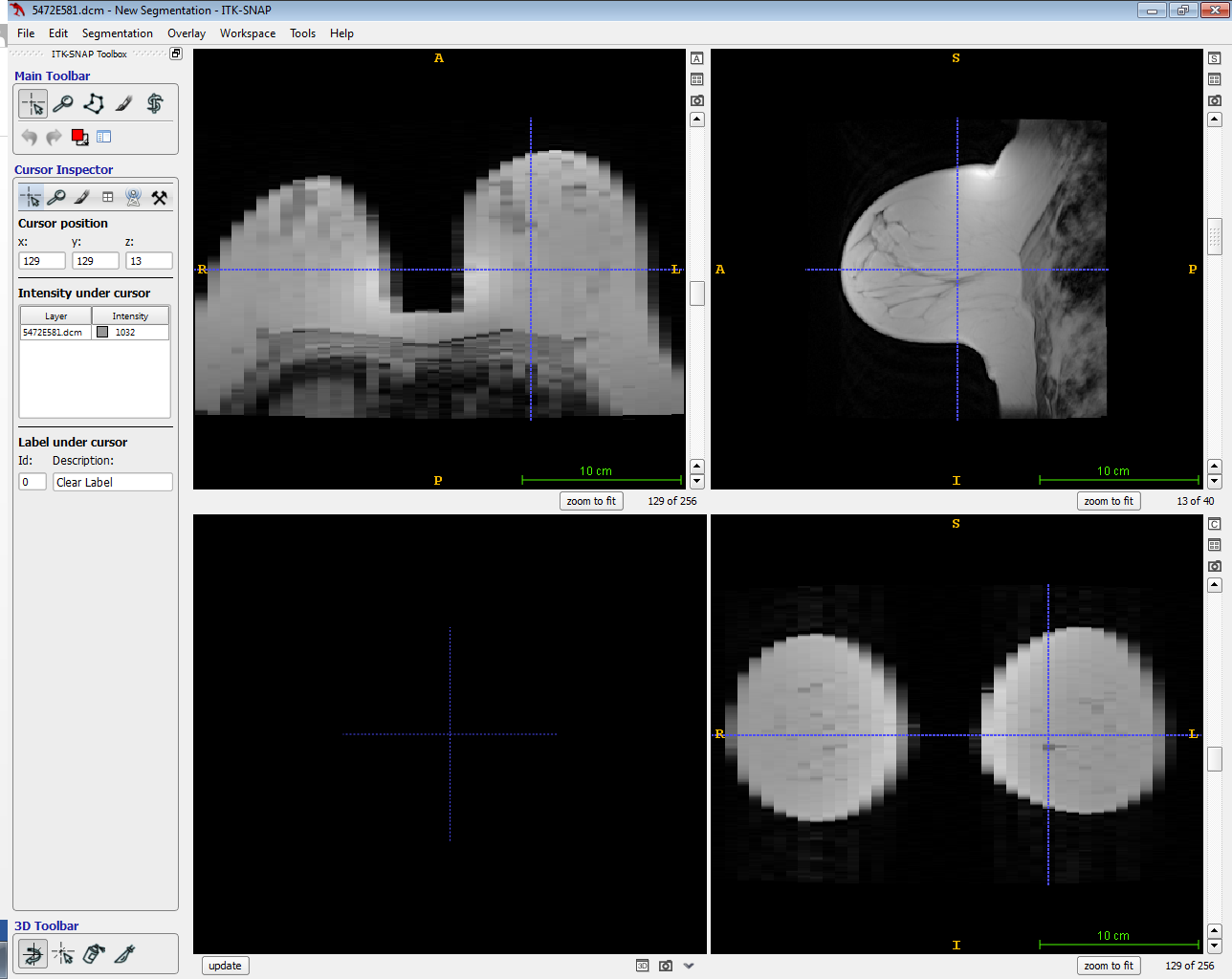


The back of the breast should be up to the retromammary space or pectoral muscle, which is a dark mass on the MRI images.


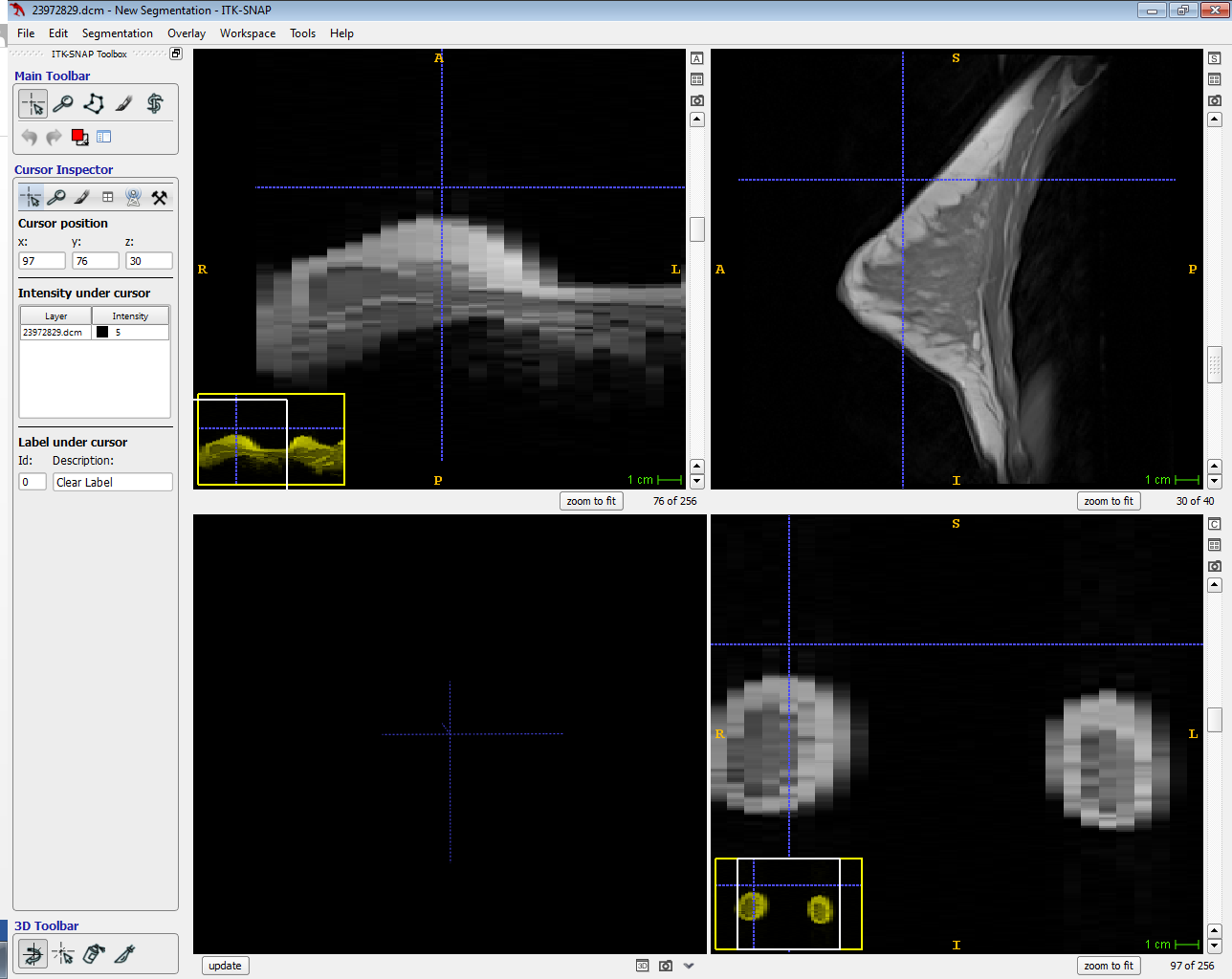


Retromammary space

Where the retromammory space or pectoral muscle is not visible, the mask should go back to the edge of the MRI image.


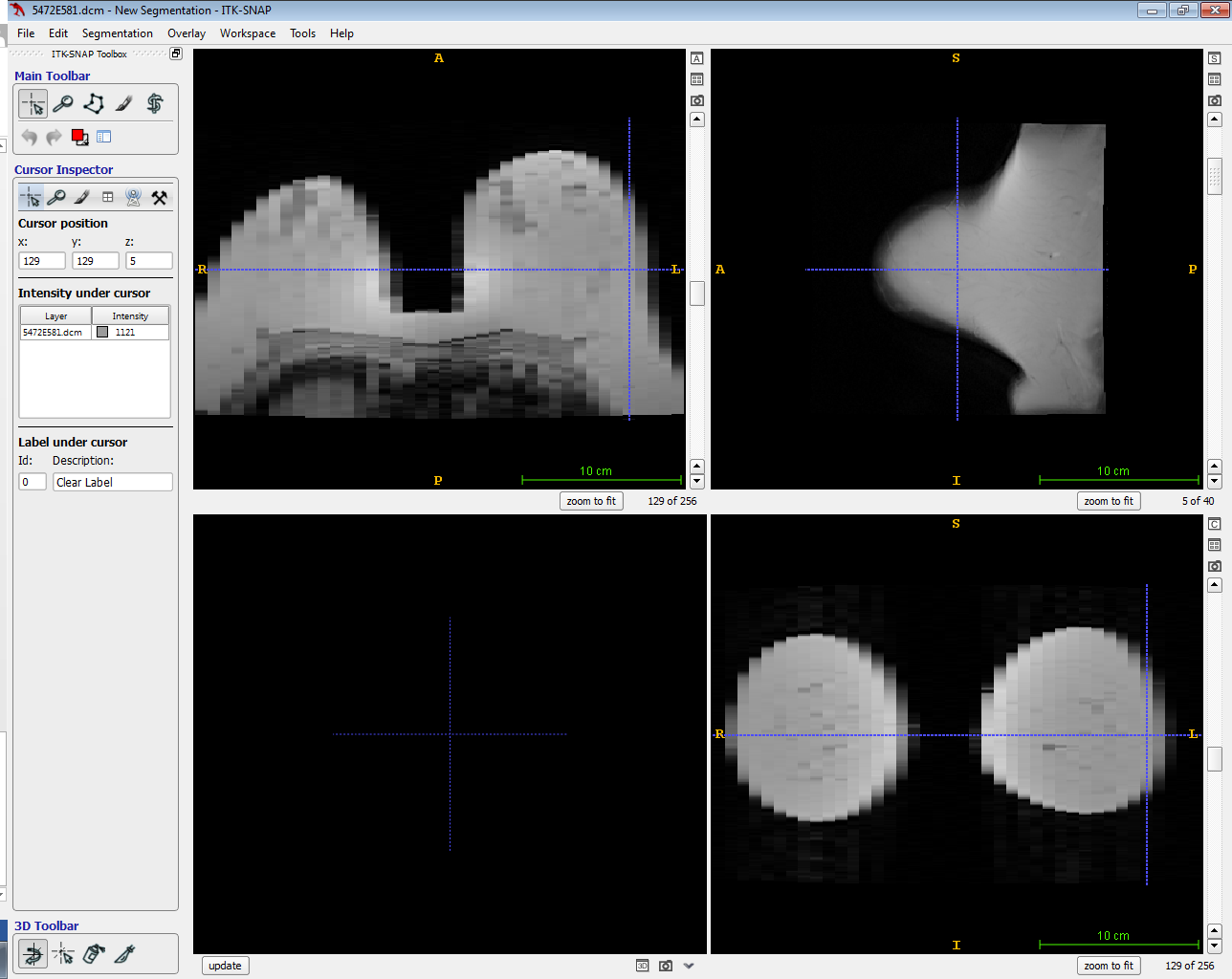


The white space behind the pectoral muscle should not be included in the mask. Where the contour of the breast is hazy, as in the image below, ensure to include all white areas.


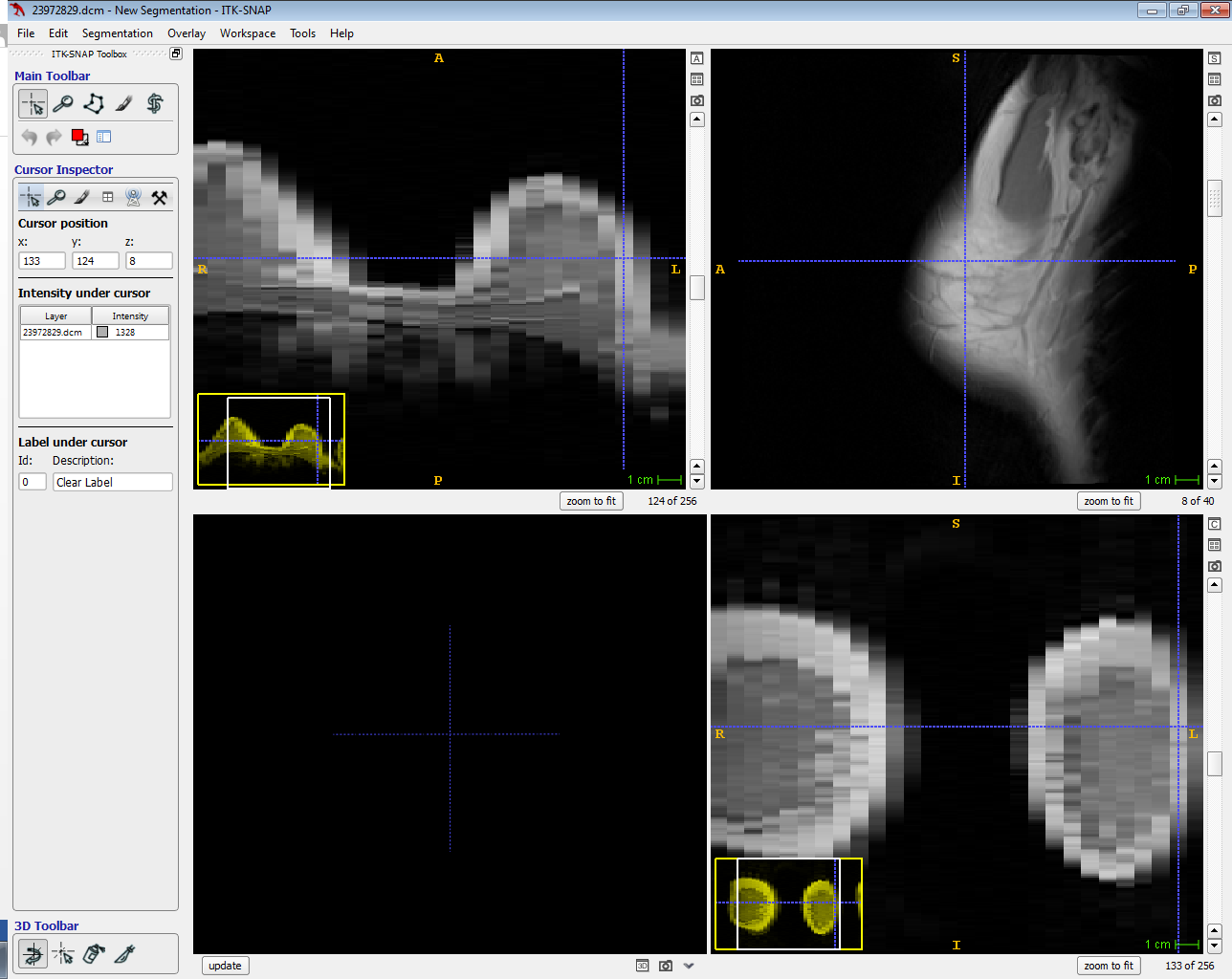


Retromammary space

Pectoral muscle

Exclude lateral images where the breast is very faded or appears to be floating as in the images below.


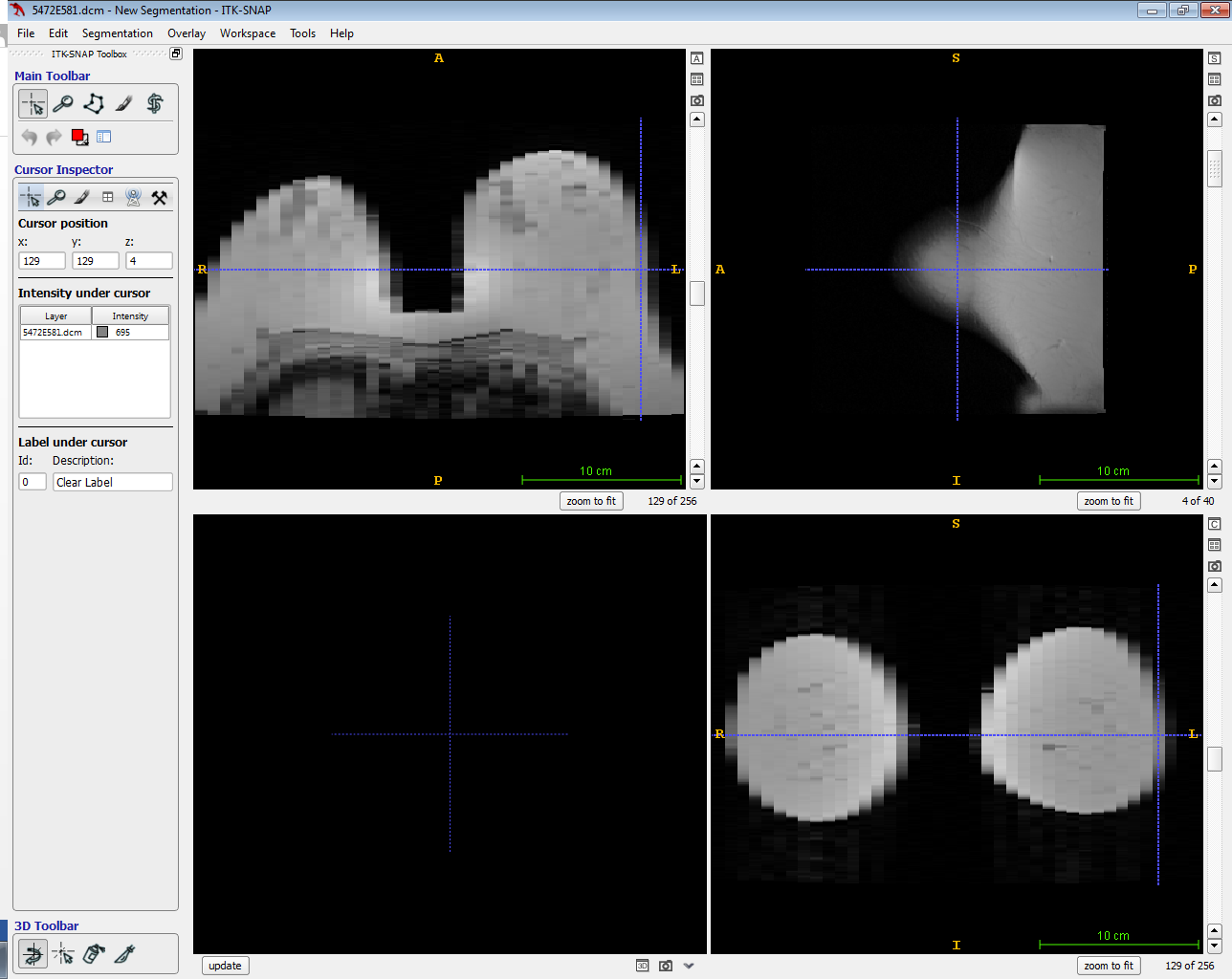

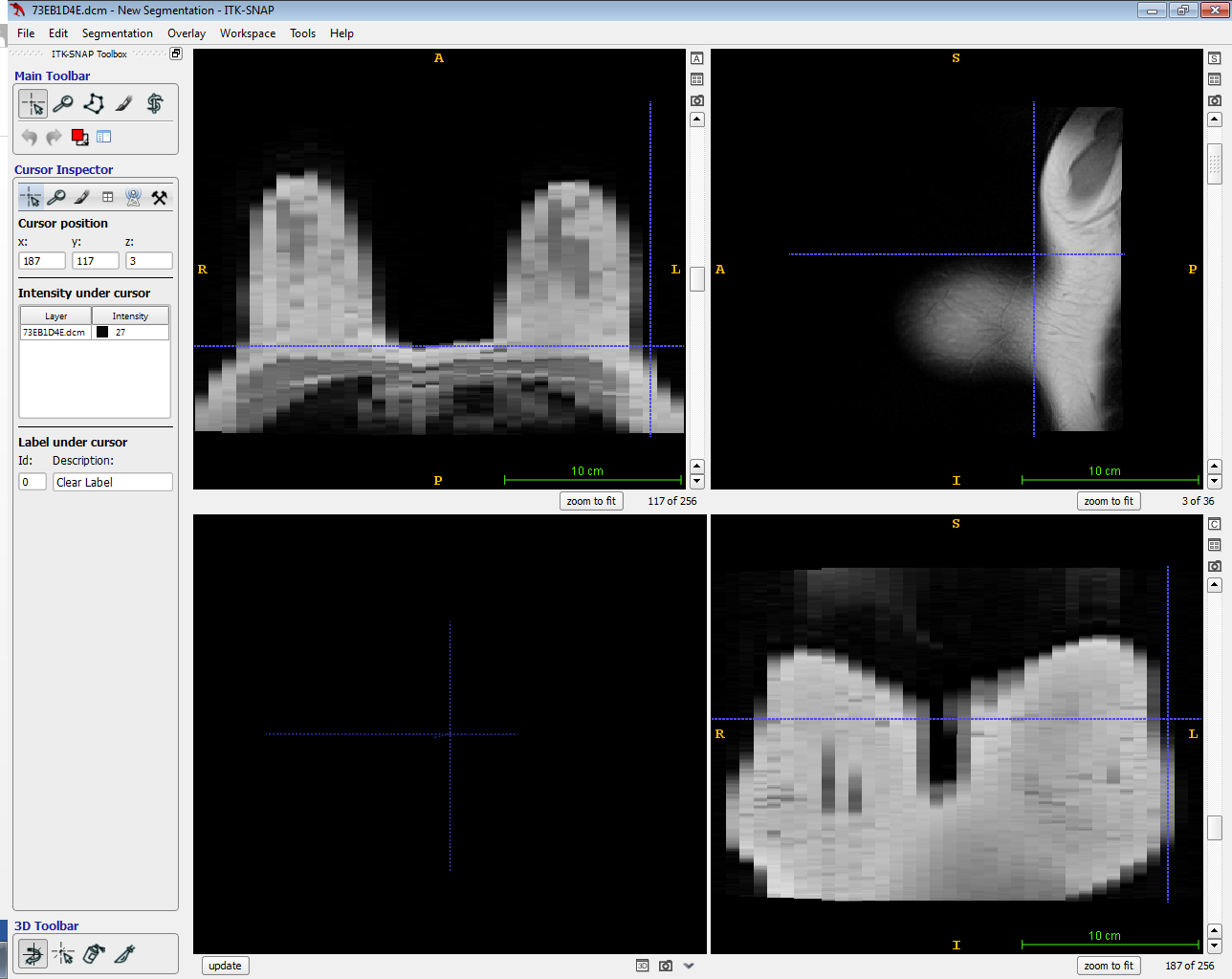

Supplement: Supplementary file 1 — Appendix S1. Data availability statement. Appendix S2. Statistical and epidemiological analysis. Figure S1. Exemplar MR images from a single subject, illustrating the different spatial resolution and contrast in the various image types acquired. Figure S2. Concepts involved in the heuristic algorithms of the BC‐FCM refinement algorithm. Figure S3. Distribution of breast volumes and percentage water as measured by the different segmentation and fat‐water estimation methods. Nomenclature for method names is as described in the main text. Figure S4. Results of Bland‐Altman analysis of (A) breast volume measurements and (B) percentage water measurements obtained using different segmentation methods. Nomenclature of method names is as described in the main text. Table S1. Dice and Jaccard coefficients obtained by comparing manual and automatically segmented masks. Table S2. Dice and Jaccard coefficients obtained by comparing manual and automatically segmented masks for five representative cases in which the high‐resolution T1‐w datasets were fully manually segmented. Appendix S3. MRI manual masking protocol. [file MP-44-4573-s001.docx]
