# Supplementary material for: Breast MRI segmentation for density estimation: Do different methods give the same results and how much do differences matter?
Source: Med Phys. 2017 Jul 25;44(9):4573–92. doi: 10.1002/mp.12320 (PMC5697622; doi:10.1002/mp.12320)
Supplement: Supplementary file 2 [file MP-44-4573-s002.docx]

Additional Supporting Information may be found online in the supporting information tab for this article.

**Appendix S1.** Data availability statement.

**Appendix S2.** Statistical and epidemiological analysis.

**Figure S1.** Exemplar MR images from a single subject, illustrating the different spatial resolution and contrast in the various image types acquired.

**Figure S2.** Concepts involved in the heuristic algorithms of the BC-FCM refinement algorithm.

**Figure S3**. Distribution of breast volumes and percentage water as measured by the different segmentation and fat-water estimation methods. Nomenclature for method names is as described in the main text.

**Figure S4.** Results of Bland-Altman analysis of (A) breast volume measurements and (B) percentage water measurements obtained using different segmentation methods. Nomenclature of method names is as described in the main text.

**Table S1.** Dice and Jaccard coefficients obtained by comparing manual and automatically segmented masks.

**Table S2.** Dice and Jaccard coefficients obtained by comparing manual and automatically segmented masks for five representative cases in which the high-resolution T1-w datasets were fully manually segmented.

**Appendix S3.** MRI manual masking protocol.
